# Boronic Acids and Their Derivatives in Medicinal Chemistry: Synthesis and Biological Applications

**DOI:** 10.3390/molecules25184323

**Published:** 2020-09-21

**Authors:** Mariana Pereira Silva, Lucília Saraiva, Madalena Pinto, Maria Emília Sousa

**Affiliations:** 1 CIIMAR, Laboratório de Química Orgânica e Farmacêutica, Departamento de Ciências Químicas, Faculdade de Farmácia, Universidade do Porto, Rua de Jorge Viterbo Ferreira n° 228, 4050-313 Porto, Portugal; up201909171@ff.up.pt (M.P.S.); madalena@ff.up.pt (M.P.); 2 LAQV/REQUIMTE, Laboratório de Microbiologia, Departamento de Ciências Biológicas, Faculdade de Farmácia, Universidade do Porto, Rua de Jorge Viterbo Ferreira n° 228, 4050-313 Porto, Portugal

**Keywords:** boronic acids, biological applications, synthesis of boronic acid derivatives, boron-containing compounds

## Abstract

Boron containing compounds have not been widely studied in Medicinal Chemistry, mainly due to the idea that this group could confer some toxicity. Nowadays, this concept has been demystified and, especially after the discovery of the drug bortezomib, the interest for these compounds, mainly boronic acids, has been growing. In this review, several activities of boronic acids, such as anticancer, antibacterial, antiviral activity, and even their application as sensors and delivery systems are addressed. The synthetic processes used to obtain these active compounds are also referred. Noteworthy, the molecular modification by the introduction of boronic acid group to bioactive molecules has shown to modify selectivity, physicochemical, and pharmacokinetic characteristics, with the improvement of the already existing activities. Besides, the preparation of compounds with this chemical group is relatively simple and well known. Taking into consideration these findings, this review reinforces the relevance of extending the studies with boronic acids in Medicinal Chemistry, in order to obtain new promising drugs shortly.

## 1. Introduction

The use of boron in the design of drugs is fairly recent and most biological activities of these compounds have been reported over the last decade [1]. For a long time, compounds with boron were put aside in studies of Medicinal Chemistry because they were thought to be toxic, mainly because of their use in ant poisoning. Nowadays, this believe is demystified, and boron-containing compounds are usually considered as non-toxic [2].

**Figure 1:**
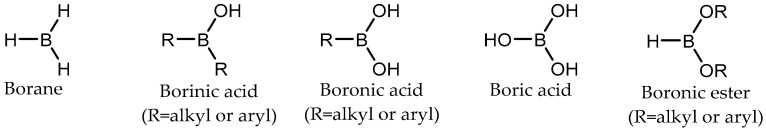
Organoboron compounds.

Boron compounds are found in nature in high concentrations, mainly in vegetables, fruits, and nuts, and the boron natural derivative boric acid is even used as a preservative in eyewash and as a buffer in biological assays [2]. There are many organoboron compounds (Figure 1) but, in organic chemistry, boronic acid is the most commonly studied boron compound [2].

Boronic acids were first synthetized in 1860 by Edward Frankland [3] and can be used as building blocks and synthetic intermediates [1,4]. The application of these compounds in synthetic chemistry is due to their versatile reactivity, stability, and low toxicity [5,6,7] and also because when considering drug design boronic acid is further degraded to boric acid, a “green compound”, being the latter eliminated by the body [2,5,8].

Boronic acids have unique physicochemical and electronic characteristics although boron has interesting similarities with carbon. Besides, boronic acids are also considered bioisosteres of carboxylic acids [7].

**Figure 2:**
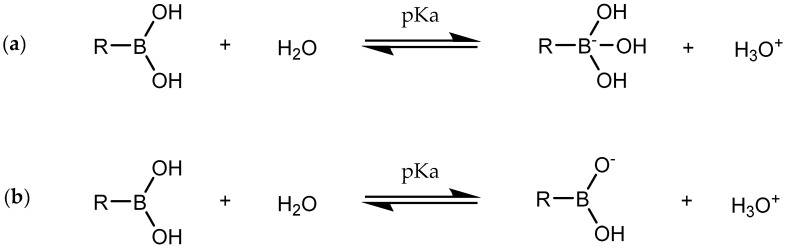
Ionization equilibrium of boronic acid in aqueous solutions. (**a**) Boronic acid as Lewis acid. (**b**) Boronic acid as Brønsted–Lowry acid.

Boronic acids are considered Lewis acids (Figure 2a), having a p*Ka* value of 4–10 [2,4,5,9]. These values can vary depending on the substituents on the boronic acids derivatives [4,9]; usually aryl boronic acids are more acidic than alkyl boronic acids [5]. If an electron-withdrawing group is attached to an aromatic boronic acid, the p*Ka* value decreases, while an electron-donating group increases the p*Ka* value [5,10].

At physiological pH, boronic acids remain at their protonated uncharged trigonal form but, in aqueous solutions with pH values higher than p*Ka*, they are converted to anionic tetrahedral forms [2,4,6,7]. Therefore, the equilibrium between the ionized and unionized form depends on differences between pH level and p*Ka* value of the boronic acid. Only in rare cases, boronic acids are considered Brønsted–Lowry acids (Figure 2b); this occurs when the formation of the tetrahedral form is unfavorable [5].

As Lewis acids, these compounds can form complexes with Lewis bases like hydroxide anions, and electron-donating groups, like nitrogen or oxygen and also behave as electrophiles [1,2,8,10]. Therefore, they can form a reversible non-ionic bond with nucleophilic biological compounds such as enzyme residues, nucleic acids, or hydroxyl groups from carbohydrates [2]. These characteristics are very interesting for the creation of possible compounds containing boronic acid that could be used for therapeutic applications as follows.

**Figure 3:**
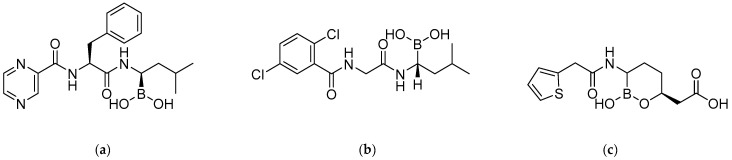
Drugs containing boronic acid approved by the Food and Drug Administration (FDA) and the European Medicines Agency (EMA). (**a**) Bortezomib. (**b**) Ixazomib. (**c**) Vaborbactam.

Bortezomib (Figure 3a), also known as Velcade, was the first boronic acid-containing drug approved by Food and Drug Administration (FDA) in 2003 and by European Medicines Agency (EMA) in 2004. This drug is a proteasome inhibitor, used for treatment of multiple myeloma [2,7,11]. Due to the overall desirable features of boronic acids and since the approval of Velcade, interest for boronic acids in Medicinal Chemistry has been arising throughout the years, leading to the discovery of two more medications approved by drug authorities—ixazomib and vaborbactam. Ixazomib (Figure 3b), a *N*-dipeptidyl boronic acid approved by FDA and EMA in 2015 and 2016, respectively, is also used for treatment of multiple myeloma, having the same mechanism of bortezomib [12,13,14]. Vaborbactam (Figure 3c) was approved by FDA in 2017 and by EMA in 2018. This cyclic boronic acid, a β-lactamase inhibitor, has been used in combination with antibiotics for the treatment of urinary, abdominal, and lung infections [15,16,17].

Since the approval of these drugs, many biological applications have been reported over the last years for different boronic acid derivatives. Several reviews have addressed in depth their syntheses [18,19,20,21] and pharmaceutical applications [1,2,4,6,7,8,22].

The main objective of this review is to provide a broad overview and update on the Medicinal Chemistry of boronic acids, as well of some of their derivatives, emphasizing studies of structure–activity relationships and other applications beyond pharmaceuticals. Furthermore, we expect that this review will increase the interest of medicinal chemists to this privileged motif.

## 2. Synthesis and Biological Applications of Boronic Acids Derivatives

Boronic acid is a stable and generally a non-toxic group that is easily synthetized and, due to these features, can be used in several synthetic reactions including metal-catalyzed processes like Suzuki–Miyaura reaction, acid catalysis, asymmetric synthesis of amino acids, and hydroboration. Because of this, organoboron compounds are extensively used as building blocks intermediates in organic chemistry reactions [23,24,25,26,27]. One of the most noticeable applications of these compounds is the cross-coupling metal-catalyzed Suzuki–Miyaura, where they are used in order to form new C-C bonds. This is a complex reaction that involves steps such as oxidative addition, ligand substitution, transmetalation, and reductive elimination [23,24].

**Scheme 1:**
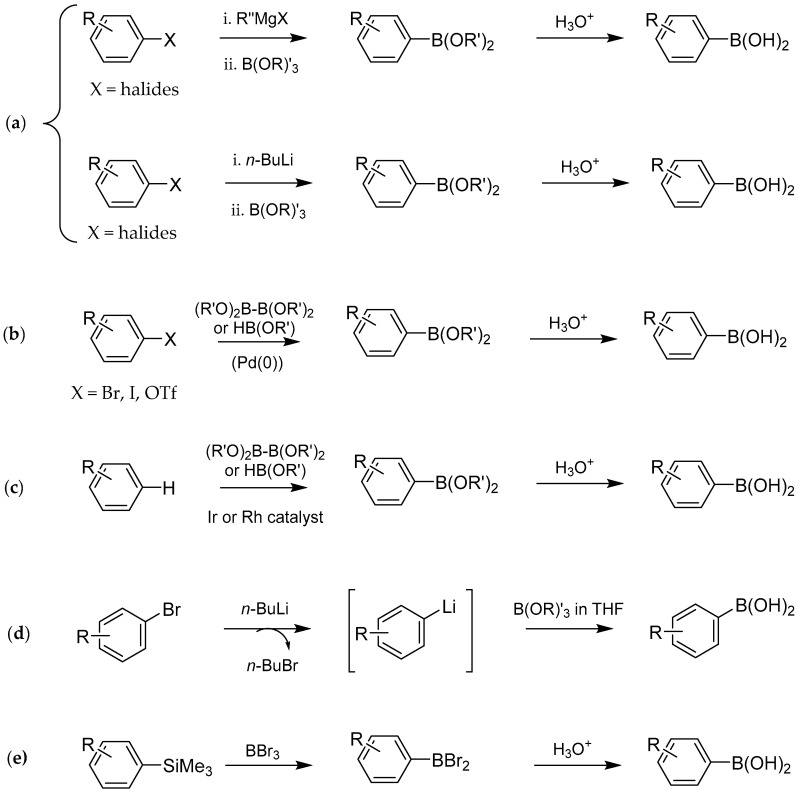
Synthetic processes to obtain aryl boronic acids. (**a**) Electrophilic trapping of arylmetal intermediates with borate esters from aryl halides using Grignard reagents or through lithium–halogen exchange, respectively. (**b**) Coupling of aryl halides with diboronic acid reagents. (**c**) Direct boronylation by transition metal-catalyzed aromatic C–H functionalization. (**d**) Bromide–lithium exchange reaction through flow chemistry. (**e**) Transmetallation of aryl silanes and stannanes.

When in drugs, boronic acids are mostly present in the form of aryl boronic acids. Besides this form, heterocycles containing boronic acids, such as pyridinyl, pyrrolyl, and indolyl derivatives, are also very useful in Medicinal Chemistry and their syntheses is accomplished in a similar fashion of aryl boronic acids [5]. The process of transmetalation to palladium(II) halides is a crucial step in cross-coupling reactions of organoboron compounds [28]. One of the first and most common methods to synthetize phenylboronic compounds was through electrophilic trapping of arylmetal intermediates with borate esters at low temperature (Scheme 1a). This reaction usually involves the addition of a methylborate to a solution of phenylmagnesium bromide but unfortunately results in low yields [5,29]. Besides using a Grignard reagent, in this synthetic pathway, boronic acids can also be prepared through lithium–halogen exchange (Scheme 1a) [30,31]. Aryl boronic acids can also be obtained through transmetallation of aryl silanes and stannanes, transition metal-catalyzed coupling of aryl halides with diboronic acid reagents and for direct boronylation by transition metal-catalyzed aromatic C–H functionalization [5].

The coupling of aromatic halides of diboronic acid reagents (Scheme 1b) was discovered by Miyaura et al., where diboronyl esters like diborylpinacolate (B_2_pin_2_) undergo a cross-coupling reaction with arylbromides, iodides, and triflates under palladium catalysis. A limitation of this reaction is the overwhelming price of diboronic acid reagents, justifying the use of a catalyst like palladium (II) chloride (PdCl_2_). Alternatively, the diboronic acid reagent could be replaced by pinacolborane [5,32]. Direct boronylation of phenyl rings (Scheme 1c) can be used for the synthesis of phenylboronic acids as an atom-economy method. In this reaction, diboronyl esters of dialkoxyboranes can be used as diboronic acid reagents and a catalyst, for instance iridium or rhodium, is needed for producing the desired product [33,34]. More recently, it has been proved that it is possible to synthetize boronic acids by using flow chemistry (Scheme 1d). Through bromine–lithium exchange reactions and by successfully suppressing the protonation and butylation side reactions through flow chemistry, it was possible to prioritize the borylation reaction and, therefore, the synthesis of a phenylboronic acid was accomplished [35]. In the case of transmetallation of aromatic silanes and stannanes (Scheme 1e), trialkylaryl silanes and stannanes are transmetallated with a boron halide, resulting in an arylboron dibromide compound that then undergoes acidic hydrolysis, resulting in the formation of relatively simple phenylboronic acids [5,36].

**Figure 4:**
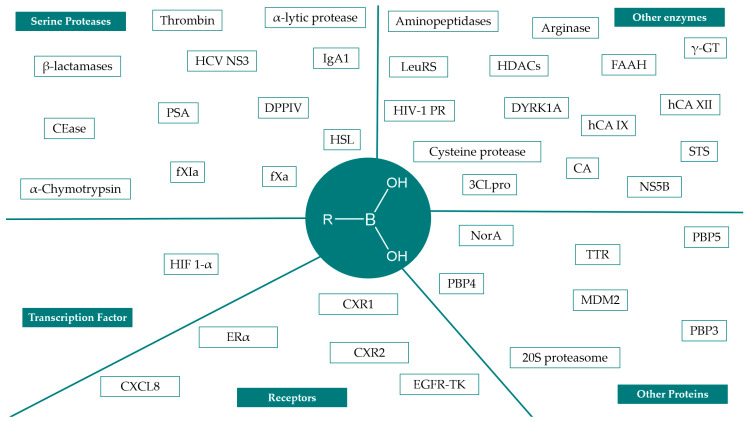
Molecular targets of boronic acids. Serine Proteases include: Dipeptidyl Peptidase IV (DPPIV), Factor Xa (fXa) and XIa, Hepatitis C Virus (HCV) NS3, Hormone-sensitive lipase (HSL), IgA1 protease, Pancreatic cholesterol esterase (CEase), Prostate specific antigen (PSA), Thrombin, α-Chymotrypsin, α-lytic protease, β-lactamases. Other enzymes include: Aminopeptidases, Arginase, human Carbonic anhydrase IX (hCA IX), hCA XII), Cysteine protease, Fatty Acid Amide Hydrolase (FAAH), Histone deacetylases (HDACs), HIV-1 Protease (HIV-1 PR), Leucyl-tRNA synthetase (LeuRS), RNA-dependent RNA polymerase NS5B (NS5B), Tyrosine Kinase DYRK1A (DYRK1A), Steroid sulfatase (STS), γ-Glutamyl transpeptidase (γ-GT), 3Clike protease (3CLpro). Proteins include: Murine double minute 2 (MDM2), NorA efflux pump (NorA), Penicillin binding protein 3 (PBP3), PBP4, PBP5, Transthyretin (TTR), 20S proteasome. Transcription factors include: Hypoxia-inducible factor 1-α (HIF 1-α). Receptors include: Chemokine receptors 1 (CXCR1), CXCR2, CXCL8, Epidermal growth factor receptor tyrosine kinase (EGFR-TK), Estrogen Receptor α (ERα).

As previously mentioned, over the years, boronic acids have proved to be very interesting compounds in Medicinal Chemistry, due to their overall characteristics and various biological applications and potential molecular targets. In Figure 4, the general molecular targets already described for boronic acids are represented [1,4,7,22]. Table 1 shows some of the clinical trials that are ongoing or have been already completed with boronic acids and other Boron containing compounds.

In this review some of the molecular targets of boronic acid-containing compounds mentioned in Figure 4 will be addressed with more detail, being the sections of the review divided according to the type of therapeutic application of boronic acid derivatives.

### 2.1. Anticancer Activity

Cellular homeostasis is accomplished through deterioration of intracellular proteins. This is possible due to the action of the essential ubiquitin-proteasome pathway [1,4,37]. This pathway is involved in the regulation of cell-cycle and apoptosis through stabilization of proteins such as the tumor suppressor p53 [1,37], making proteasome a promising target for cancer therapy [1,4,37,38]. 

As mentioned before, bortezomib is the first in-class proteasome inhibitor used in the treatment of multiple myeloma [1,2,7,37]. Bortezomib drug design was based on the first synthetic peptide proteasome inhibitors, the aldehyde proteasome inhibitors. It was demonstrated that the replacement of the aldehyde moiety with boronic acid led to the resolution of problems associated with these peptide aldehyde inhibitors, for instance, the rapid dissociation from proteasome, the inactivation by oxidation, the lack of specificity for proteasome and the unfavorable pharmacokinetics. Besides, the boronic acid group significantly increased the potency of the proteasome inhibitor [38,39,40]. The reason why boronic acid was added in the drug design of this compound was due to the known high selectivity and low dissociation rates of this group towards the active site of proteasome. It is known that the boronic acid moiety of bortezomib forms a complex with the nucleophilic *N*-terminal threonine hydroxyl group, present in the active site of the proteasome, leading to the disruption of the protein complex NF-γB, and ultimately promoting growth inhibition and apoptosis of cancer cells [7,41].

Besides bortezomib, other boronic acid compounds have also shown activity as proteasome inhibitors. Chalcones are a well-established class of anticancer agents that induce apoptosis in many types of cancer cells, including breast cancer. One limitation of chalcones is related to the carboxylic analogues that do not reveal selective toxicity towards malignant epithelial breast cells, also targeting healthy cells [42]. One approach for breast cancer therapy is through inhibition of mouse double minute 2 (MDM2) oncoprotein that is overexpressed in these cancer cells [42]. MDM2 is capable of negatively regulate p53 and is therefore responsible for blocking p53 tumor suppression activity [42,43]. The development of irreversible and selective chalcones is an interesting approach for breast cancer therapy. Since the carboxylic acid group binds near the Lysine51 (Lys51) of MDM2, and being boronic acid bioisosteres, the latter were proposed as promising to be associated with chalcones, which could lead to a selective targeting [42]. The donating amino group of Lys51, present in MDM2, is linked to the boronic acid scaffold, and this binding is possibly stronger than when carboxylic acid is present. At the same time occurs the break of the salt bridge with glutamic acid25 (E25) of MDM2, which results in a stronger selective inhibition of MDM2 in cancer cells and, consequently, growth arrest attributable to the boronic acid group. The replacement of carboxylic acid group by boronic acid (compounds **2**, **4**, and **5**) revealed an increase in selective toxicity towards breast cancer cells (results in Table 2) and also that these boronic acid-containing chalcones are more toxic towards these cells than any other known chalcones [42].

In addition to the chalcone-boronic acid derivatives **2**, **4**, and **5**, other researchers [37] have also studied the replacement of carboxylic acid by boronic acid in chalcones. Two compounds, AM 58 (**3**) and AM 114 (**8**) were synthetized and, revealed cytotoxic activity against human colon carcinoma cells (HCT116 p53+/+ cells: **3**, half maximal inhibitory concentration (IC_50_) of 3.85 µM; **8**, IC_50_ of 1.49 µM) by inhibiting the 20S proteasome, which consequently resulted in an agglomeration of some ubiquitinated proteins and of p53. It is thought that AM 58 (**3**) and AM 114 (**8**) interrupt the MDM2-p53 interaction through connection to the binding site [37].

**Scheme 2:**
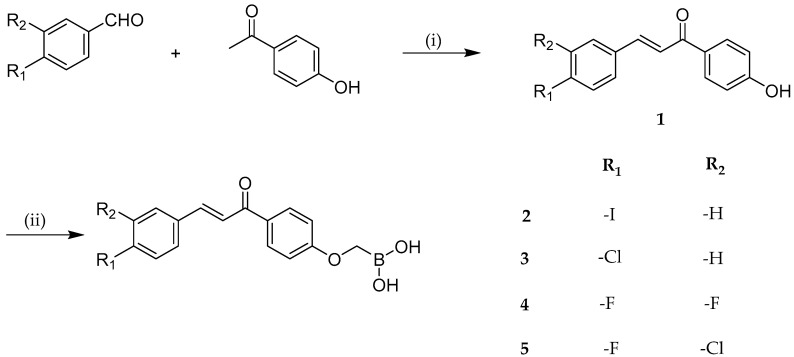
Synthesis of the chalcone-boronic acid compounds **2**–**5**. Reaction conditions: (**i**) KOH, MeOH, reflux, 0 °C to room temperature (r.t.), 55 min; (**ii**) (1) NaH, pinacol (bromomethyl)boronate, THF, 0 °C to r.t., overnight; (2) NaOH, H_2_O.

**Scheme 3:**
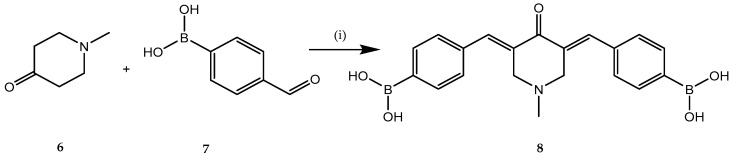
Synthesis of the chalcone-diboronic acid compound **8**. Reaction conditions: (**i**) KOH, EtOH, H_2_O, 0 °C to r.t., overnight.

The synthesis of compounds **4**–**8** is based on Claisen–Schmidt aldol condensation [37,42]. In the case of compounds **2**–**5** (Scheme 2), the intermediate that is formed (**1**) is treated with pinacol (bromomethyl)boronate and sodium hydride (NaH) in the presence of DMSO (dimethyl sulfoxide). The process of deprotection occurs in alkaline conditions [37]. Compound **8** was prepared in a different way (Scheme 3). A solution of methyl-piperidinone (**6**, 1 eq.) was mixed with a formylphenylboronic acid (**7**, 3 eq.) in presence of EtOH and KOH (6 eq.) [37].

**Scheme 4:**
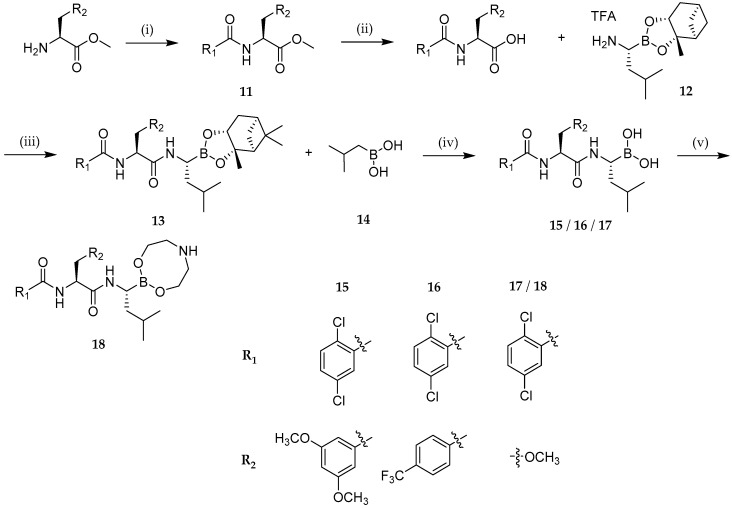
Synthesis of the dipeptide boronic acids **15**–**17**, and of the prodrug **18**. Reaction conditions: (**i**) R_1_COOH, 1-ethyl-3-(3-dimethylaminopropyl)-carbodiimide hydrochloride (EDCI), 1-hydroxybenzotriazol (HOBt), N,N-diisopropylethylamine (DIPEA), CH_2_Cl_2_, −10 °C to r.t.; (**ii**) (1) LiOH·H_2_O, MeOH, H_2_O, r.t.; (2) 1 N HCl, EtOAc, r.t.; (**iii**) EDCI, HOBt, DIPEA, CH_2_Cl_2_, −10 °C to r.t.; (**iv**) 1 N HCl, MeOH, n-hexane, r.t., 22 h; (**v**) EtOAc, diethanolamine (DEA), 74 °C to r.t., overnight.

Dipeptidyl boronic acids are common groups used for the design of proteasome inhibitors due to the bioactivity of bortezomib [1]. A tridimentional-quantitative structure–activity relationship (3D-QSAR) study of bortezomib allowed the synthesis of compounds with promising proteasome inhibitory activity [40]. The 3D-QSAR studies revealed that there are three important regions for the interaction between bortezomib and proteasome: (i) A covalent site to allow the bonding between bortezomib and proteasome Thyr1 residue; (ii) an aromatic ring; (iii) a pyrazinyl as an acceptor and hydrophobic group. Based on these results, a series of dipeptide boronic acid compounds were synthesized, being compounds **15** and **16** (Scheme 4) the most promising ones. Compound **15** has a better IC_50_ value than bortezomib (**15**, IC_50_ of 4.60 nM; bortezomib, IC_50_ of 7.05 nM) and can be considered a lead compound. The studies of the mechanism of action indicated that this dipeptide boronic acid proteasome inhibitor stops progression of cell cycle at G2/M phase in U266 cells, leading to growth inhibition in cancer cells. Pharmacokinetic studies in rats revealed that compound **15** can be administrated intravenously, but optimization is required to improve the concentration level of the compound at the therapeutic target [40]. 

More recently, the same group (Lei et al.) reported another study that revealed the improvement of these compounds in order to be orally administrated [44]. For that, a prodrug (**18**, Scheme 4) based on compound **17** (Scheme 4) was designed. This prodrug **18** showed both in vitro and in vivo activities (**17**, IC_50_ of 8.21 nM; **18**, IC_50_ of 6.74 nM) and has good pharmacokinetic properties [44].

In the synthesis of compounds **15** and **16** (Scheme 4), an initial step of acidic saponification of the peptide ester occurs, forming an amino acid **11**. The acid groups of the amino acid then reacted with amino boronate trifluoroacetate **12** to give dipeptidyl boronic ester **13**. The boronic ester then undergoes transesterification with isobutyl boronic acid **14**, producing compounds **15**–**17** in moderate to high yields [40]. For the synthesis of the proteasome inhibitor prodrug **18** (Scheme 4), the dipeptidyl boronic acid **17** reacted with diethanolamine (DEA), producing the desired compound with a yield of approximately 65% [44].

**Figure 5:**
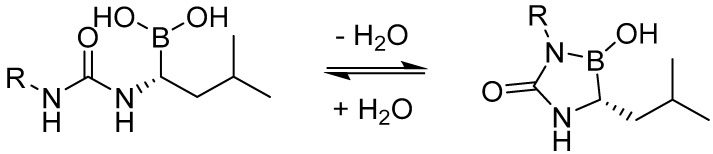
Possible formation of a cyclic boronic acid derivative, when boronic acid is located next to an urea group.

Although peptide boronic acids are a very important chemical class of proteasome inhibitors, they usually have poor pharmacokinetic properties and rapidly undergo in vivo inactivation. These results are mainly associated with instability of the peptide bonds [45]. Aiming to improve these properties, urea-containing peptide boronic acids were designed. A common strategy to improve pharmacokinetic and pharmacodynamic proprieties of peptide drugs is to replace amine or amide group by an urea scaffold. This led to the discovery of a potent compound (**25**) in different cell lines (ChT-L, IC_50_ of 0.0002 nM; HepG-2, IC_50_ of 19.38 nM; MGC-803, IC_50_ of 3.962 nM). Its potency is due to the distance between boronic acid and the urea scaffold; it is thought that low potency may occur when these groups were next to each other, allowing the formation of a cyclic boronic acid derivative (Figure 5), which happens due to the strong electrophilicity of boron atom. In terms of pharmacokinetics, compound **25** proved to have more in vivo stability than bortezomib, being this related to a higher life-time and larger volume of distribution. This compound also revealed relatively low toxicity [45].

**Scheme 5:**
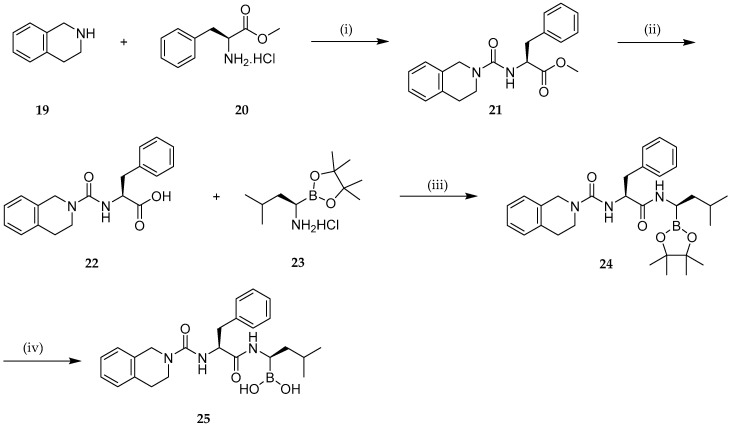
Synthesis of the urea-peptide boronic acid **25**. Reaction conditions: (**i**) CDI, dimethylformamide (DMF), MeCN, r.t.; (**ii**) (1) NaOH, 0 °C; (2) HCl, 0 °C; (**iii**) N,N′-dicyclohexylcarbodiimide (DCC), HOBt, DIPEA, CH_2_Cl_2_, r.t. (**iv**) (1) DEA, EtOAc, r.t.; (2) HCl, H_2_O, EtOAc, r.t., 2 h.

For the synthesis of compound **25** (Scheme 5), an amine derivative (**19**) and L-phenylalanine methyl ester hydrochloride (**20**) were used as initial reagents to form the urea moiety. The intermediate (**21**) was then hydrolyzed with NaOH. The α-amino boronic acid pinacol ester (**23**) was later coupled with the carboxylic acid (represented in molecule **22**), giving the α-amino boronic acid pinacol ester (**24**). Finally, the urea-containing peptide boronic acid (**25**) was obtained by elimination of the pinacol protecting group [45].

**Scheme 6:**
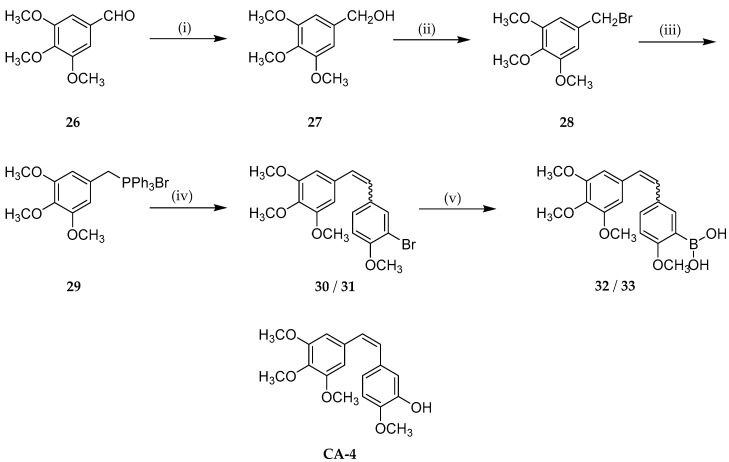
Synthesis of the cis (**32**) and trans-boronic acid (**33**) analogues based on the antimitotic agent CA-4. Reaction conditions: (**i**) NaBH_4_, MeOH, H_2_O, EtOAc, r.t., 30 min; (**ii**) PBr_3_, CH_2_Cl_2_, 0 °C to r.t., overnight; (**iii**) PPh_3_, THF, r.t., 6 h; (**iv**) 3-bromo-4-methoxybenzaldehyde, sodium bis(trimethylsilyl)amide (NaHMDS), THF, −78 °C to r.t., 6.5 h; (**v**) (1) 5% EtOAc/n-hexane, column chromatography; (2) n-BuLi, B(OMe)_3_, THF, −78 °C to r.t., overnight; (3) 3N HCl, r.t., 30 min.

The majority of boronic acid anticancer agents are proteasome inhibitors. However, besides this mechanism of action, boronic acid compounds that act on cancer targets other than proteasome have also proved to be promising. A study based on the antimitotic agent combretastatin A-4 (CA-4) [46], an agent that binds reversibly to the colchicine binding site of tubulin and prevents cellular division [46,47], was done in order to improve its solubility, without compromising its activity, since it is the low solubility in water that prevented the development of this compound as a drug [46]. Based on the fact that boronic acids could act as bioisosteres of the aromatic hydroxyl group of the C-ring of CA-4 and that at physiological pH levels, this compound remains at its unionized form (pKa 9–10), boronic acid analogues of CA-4 were synthetized. A cis and trans-boronic acid analogues (**32** and **33**, respectively) were tested and, as predicted by the docking studies, these compounds were more potent inhibitors of tubulin polymerization than CA-4. Interestingly, proliferation is dependent of the geometry of the double bond, being the analogue cis (**32**, Scheme 6) more effective (**32**, IC_50_ of 1.5 µM; **33**, IC_50_ of 7.8 µM; **CA-4**, IC_50_ of 2.0 µM). Besides these effects, no toxicity was revealed, and water solubility was enhanced, being the cis analogue (**32**) more stable and soluble than CA-4 thus allowing oral delivery. Another interesting aspect is that it is thought that the cis-boronic acid analogue **32** will not have a short plasma half-life, since the carbon–boron bond attached to the aromatic ring does not suffer hydrolysis [46].

In the synthesis of these compounds (Scheme 6), 3,4,5-trimethoxy-benzaldehyde (**26**) was reduced to give the alcohol (represented in molecule **27**) that was treated with phosphorus tribromide yielding the corresponding benzyl bromide (represented in molecule **28**) that subsequently was transformed into a phosphonium salt (**29**). A Wittig coupling allowed the transformation of this salt into cis and trans stilbenes (**30** and **31**) that were separated through flash column chromatography. After this separation, the isomers were lastly converted into the desired cis and trans-boronic acids [46].

Later, the same group [46,47] developed two chalcone analogues of compound **32** (**36** and **38**) and their effects on tubulin polymerization and growth inhibition were evaluated, in various human cancer cell lines [47]. From these compounds, **36** was the most potent one. Interestingly, this analogue revealed no inhibition on tubulin polymerization, but proved to have antiproliferative activity against different cell lines at micromolar and nanomolar concentrations (Table 3) and also revealed angiogenesis inhibition at 5 μΜ concentration. In fact, this anti-angiogenic activity is very promising, since the compounds that target cancer vascular cells usually do not demonstrate drug resistance comparing to cancer epithelial cells [47].

**Scheme 7:**
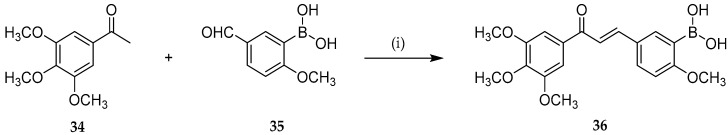
Synthesis of the chalcone-boronic acid analogue **36**. Reaction conditions: (**i**) (1) 1-(3,4,5-trimethoxyphenyl)ethenone, 5-formyl-2-methoxyboronic acid, NaOH, r.t., overnight; (2) H_2_O, HCl.

**Scheme 8:**
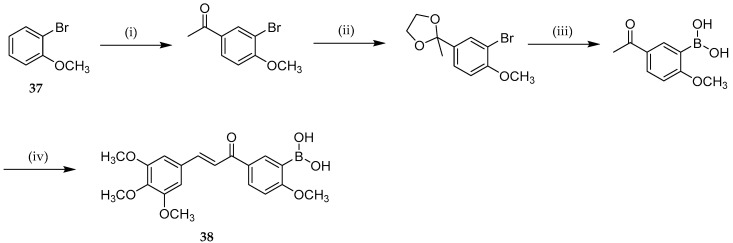
Synthesis of the chalcone-boronic acid analogue **38**. Reaction conditions: (**i**) CH_2_Cl_2_, AlCl_3_, acetic anhydride, r.t. to 40 °C, 1 h; (**ii**) Ethylene glycol, benzene, reflux, PTSA; (**iii**) (1) THF, n-BuLi, B(OMe)_3_, −78 °C, 90 min; (2) HCl, r.t., 30 min; (**iv**) (1) 3,4,5-trimethoxybenzaldehyde, NaOH, r.t., overnight; (2) H_2_O, HCl.

Chalcone analogues **36** and **38** were synthetized through Claisen–Schmidt condensation under alkaline conditions [47]. For compound **36** (Scheme 7), condensation between a ketone (**34**) and a substituted aldehyde (**35**) occurred. In the case of compound **38** (Scheme 8), a Friedel–Crafts acylation with 1-bromo-2-methoxybenzene (**37**) occurred firstly followed by ketone deprotection and a final step of Claisen–Schmidt condensation [47].

**Scheme 9:**
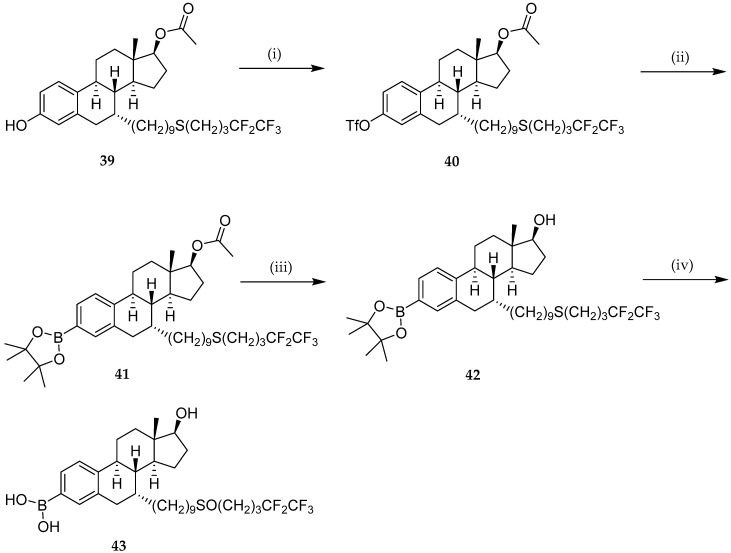
Synthesis of a SERD boronic acid analogue **43** based on the drug fulvestrant. Reaction conditions: (**i**) Triflic anhydride, pyridine, CH_2_Cl_2_, −10 °C; (**ii**) B_2_pin_2_, palladium(II) acetate (Pd(OAc)_2_), tricyclohexylphosphine, MeCN, 80 °C; (**iii**) KOH, MeOH/THF, 0 °C to r.t., 4 h; (**iv**) mCPBA, CH_2_Cl_2_, 0 °C.

A boronic acid (**43**, Scheme 9) based on commercially available fulvestrant was designed and synthetized to improve the bioavailability of this drug, so it could be administrated orally and not by intramuscular injection. Fulvestrant is a selective estrogen receptor down-regulator (SERD) that is indicated for estrogen receptor positive (ER+) metastatic breast cancer and acts by competitively binding to estrogen receptor α (ERα). The replacement of 3-OH group of fulvestrant by a boronic acid led to a substantial reduction of first pass metabolism, which consequently led to a significant improvement of bioavailability while maintaining the pharmacological proprieties. It is thought that boronic acid derivatives reduce the first pass metabolism due to their ability to bind covalently and reversibly to 1,2- and 1,3- diols present in sugar molecules and glycoproteins, making the compound unavailable to pass through glucuronidation. Besides improving the bioavailability of fulvestrant, compound **43** as also similar effects on cell growth inhibition in different breast cancer cell line (Table 4) [48].

The first synthetic step of to obtain this compound (Scheme 9) involves the formation of triflate of 17-acetyl S-deoxo fulvestrant (**40**) using as a starting material 17-acetyl-S-deoxo fulvestrant (**39**), followed by Miyaura borilyation reaction with bis(pinacolato)diboron in the presence of catalytic amount of palladium(II) acetate. After the removal of the acetyl group (represented in molecule **41**) under alkaline conditions, deprotection occurs through oxidation with meta-chloroperoxybenzoic acid (mCPBA), forming the desired product [48].

**Scheme 10:**
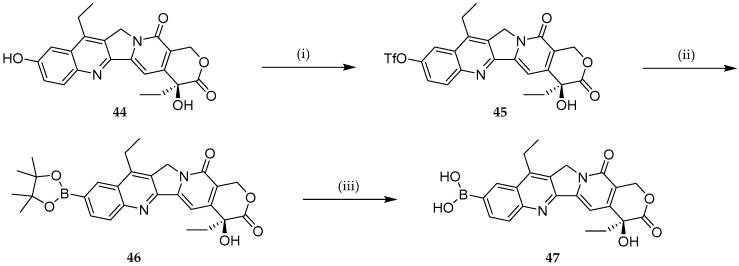
Synthesis of the camptothecin prodrug with boronic acid moiety (**47**). Reaction conditions (**i**) N,N-bis(trifluoromethylsulfonyl)aniline, triethylamine (TEA), DMF, 60 °C, 3 h; (**ii**) [1,1′-bis(diphenylphosphino)ferrocene]dichloropalladium(II) (Pd(dppf)Cl_2_), KOAc, B_2_pin_2_, 1,4-dioxane, 80 °C, 12 h; (**iii**) NaIO_4_, NH_4_OAc, 1:1 C_3_H_6_O:H_2_O, r.t., 24 h.

A strategy developed for targeting release of camptothecin, an anticancer compound that binds and subsequently inhibits the enzyme topoisomerase I, which consequently leads to cell death, was accomplished by development of a boronic acid prodrug [49]. The 7-ethyl-10-boronic acid **47** (Scheme 10) was designed based on the fact that alkyl and aryl boronic acids are easily oxidized by hydrogen peroxide (H_2_O_2_) and that usually cancer cells have increased levels of reactive oxygen species (ROS), comparing to normal cells [49,50]. Another factor that makes boronic acid prodrug **47** interesting for selective delivery of camptothecin is that the surface of cancer cells is covered with the polysaccharide glycocalyx and boronic acids can bind to the 1,2- and 1,3-diols of glycocalyx forming boronate esters, which is known to enhance drug selectivity towards cancer cells [49,51]. Through the oxidation of 10-boronic acid to 10-OH by H_2_O_2_, which is present in high levels in cancer cells, the selective release of camptothecin directly to cancer cells can be accomplished [49]. 

In the synthesis of prodrug **47** (Scheme 10), the camptothecin drug (**44**) was first converted into triflate (**45**) and then occurred a borylation of the intermediate that was mediated by palladium-catalyzed Miyaura reaction, followed by a final step of reduction of boronate ester (represented in molecule (**46**) [49].

**Figure 6:**
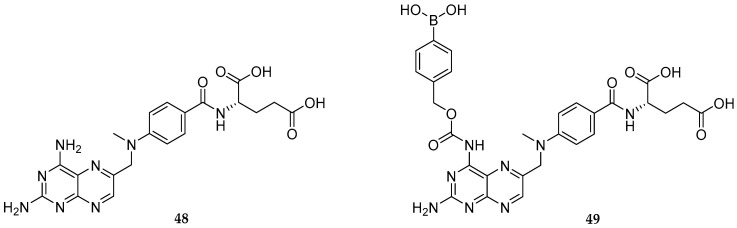
Methotrexate and its phenylboronic acid prodrug (**48** and **49**, respectively).

Similar to this approach, the phenylboronic acid prodrug **49** (Figure 6) of methotrexate (**48**, Figure 6) was designed for a more selective and effective treatment of rheumatoid arthritis (RA), since the presence of high levels of ROS in inflammatory cells is associated with RA pathology [52].

**Scheme 11:**
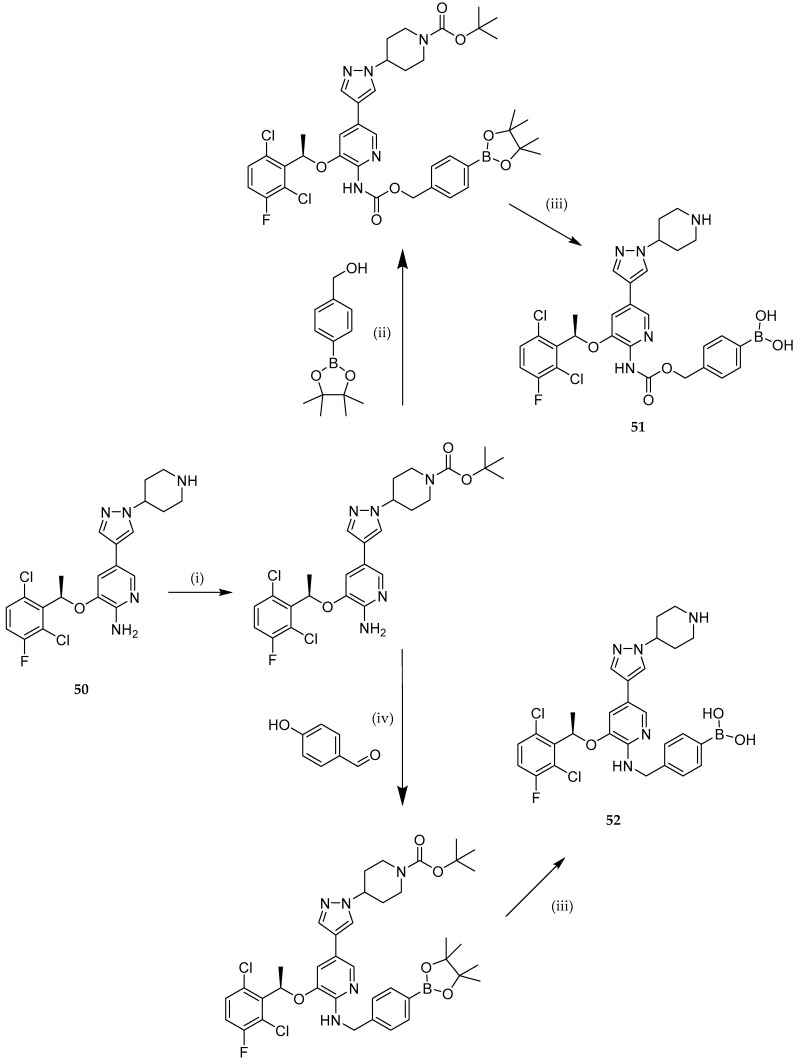
Synthesis of the crizotinib prodrugs with boronic acid moiety **51** and **52**. Reaction conditions: (**i**) Di-tert-butyl dicarbonate, THF, 12 h; (**ii**) (4-(4,4,5,5-tetramethyl-1,3,2-dioxaborolan-2-yl)phenyl)methanol, CO, Pd(OAc)_2_, copper(II) acetate monohydrate (Cu(OAc)_2_∙H_2_O), KI, DMSO, MeCN; (**iii**) TFA, CH_2_Cl_2_, r.t., 2 h (**51**) or 30 min (**52**); (**iv**) 4-hydroxybenzaldehyde, 2-picoline borane, AcOH/MeOH, overnight.

Likewise, a recent study based on the elevated levels of ROS in some cancers showed the development of a boronic acid prodrug for the selective delivery of a tyrosine kinase inhibitor drug in cancer cells. Tyrosine kinases are involved in processes of cell differentiation, proliferation, and apoptosis and are usually activated in an abnormal manner in cancer cells. One problem of some of these drugs is that they do not have cancer cells specificity, being also toxic to normal cells. This occurs with crizotinib, revealing hepatotoxicity that consequently leads to the reduction or discontinuation of the treatment with this drug. Crizotinib has a 2-aminopyridine moiety that binds to the amino acids in the ATP-binding pocket of kinases tyrosine-protein kinase Met (c-MET), ROS proto-oncogene 1 tyrosine-protein kinase (ROS1) and anaplastic lymphoma kinase (ALK). The synthesis of boronic acid prodrugs of crizotinib (**51** and **52**, Scheme 11) proved to be a good approach for reverting the problems associated with crizotinib. When present in high levels, which occurs in malignant cells, H_2_O_2_ oxidizes the boronic acid moiety and allows the selective release of crizotinib. It was also proved that, in case of prodrug **51**, more than half of the prodrug remains intact in biological conditions, therefore not releasing crizotinib [53]. 

The 2-aminopyridine of crizotinib (**50**) is modified (Scheme 11): First occurred a protection of the piperidine nitrogen with a tert-butyloxycarbonyl (Boc) group, followed by selective functionalization of the 2-aminopyridine. In the case of prodrug **51**, the moiety was introduced via carbamate, while in prodrug **52** was via alkylation, using a reductive amination approach [53].

It is thought that the autotaxin-lysophosphatidic acid axis (ATX-LPA axis) has an important role in tumor progression and metastasis. The activity of the extracellular enzyme autotaxin (ATX) is related to a threonine residue in the active site and the inhibition of this enzyme could be a good strategy for cancer therapy. Based on the hit compound 2,4-thiazolidinedione (IC_50_ of 2.50 μM), a series of SAR studies were performed in order to find a selective compound for cancer cells. A first SAR study revealed that the methoxy and carboxylic acid moieties were important for the inhibition of ATX. With the objective of improving inhibition and targeting, the carboxylic acid group was replaced by its bioisostere boronic acid **55** [41]. This replacement was based on the knowledge that carboxylic acid could bind to the active site of threonine oxygen and that boronic acid moiety in bortezomib binds to the nucleophilic oxygen lone pair of threonine [41,54]. It was proved that the introduction of boronic acid in para position significantly enhanced the inhibition of autotaxin, both in in vitro and in vivo assays, resulting in a 440-fold more active inhibitor (IC_50_ of 6 nM) [41].

**Scheme 12:**
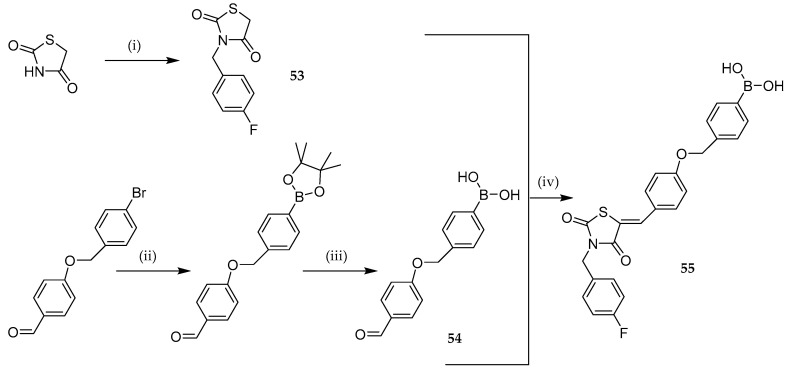
Synthesis of the boronic acid autotaxin inhibitor **55**. Reaction conditions: (**i**) NaH, DMF, 1-(chloromethyl)-4-fluorobenzene, r.t., 22 h; (**ii**) B_2_pin_2_, Pd(dppf)Cl_2_, KOAc, DMF, 80 °C, 18 h; (**iii**) THF, NaIO_4_, HCl; (**iv**) EtOH, piperidine, 22 h, reflux.

The synthesis of this compound (**55**) was performed by a convergent synthesis (Scheme 12), where the thiazolane-2,4-dione (**53**) and diphenyl boronic acid (**54**) moieties were prepared separately [41,55]. The synthesis of the diphenyl boronic acid is accomplished via Suzuki–Miyaura reaction [55]. Finally, through Knoevenagel condensation, these two moieties are linked to produce the compound **55** [41,55].

Another interesting application of boronic acid derivatives in cancer therapy is through the design of histone deacetylases (HDAC) inhibitors [56,57]. It is known that these inhibitors are associated with inhibition of cell growth, stimulation of terminal differentiation in tumor cells, and can even prevent malignant tumors formation. The design of these compounds was based on their deacetylation mechanism, acting on lysine residue of these epigenetic enzymes [56,57]. The zinc ion located in the active site of this enzyme coordinates the electrophilic carbon of the carbonyl group present in a substrate. This attack leads to the formation of a tetrahedral transition state, which is stabilized by the zinc-oxygen interaction. Since the boronic acids can act as electrophiles and be attacked by nucleophilic groups, boronic acids with α-amino acid moiety (**59**–**61**) were designed, in which boronic acid substitutes the acetamide of the acetylated lysine substrate. The boronic acid group is attacked by water in the active site, forming a transition state analogue that binds to the zinc ion, leading to the inhibition of HDAC that subsequently lead to cancer cell growth inhibition (**59**, IC_50_ of 2.0 µM; **60**, IC_50_ of 2.0 µM; **61**, IC_50_ of 1.4 µM). The α-amino acid moiety is also very important for the inhibitory activity, since its replacement decreases the potency [56].

**Scheme 13:**
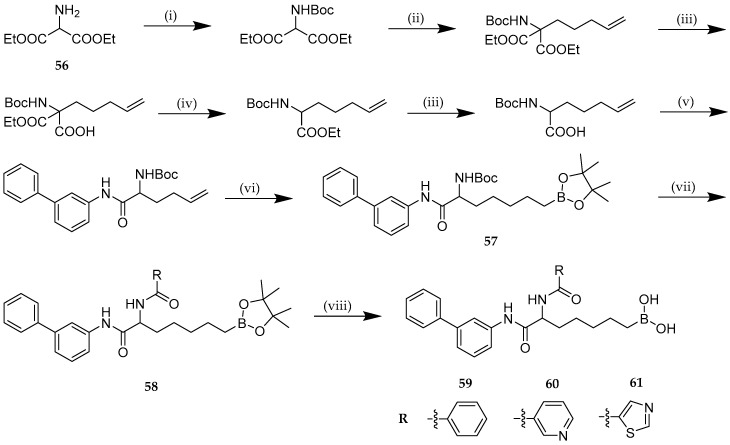
Synthesis of the boronic acids HDAC inhibitors **59**–**61**. Reaction conditions: (**i**) (Boc)_2_O, TEA, THF, r.t.; (**ii**) NaOEt, 5-Bromopent-1-ene, EtOH, reflux; (**iii**) LiOH·H_2_O, EtOH, H_2_O, 0 °C; (**iv**) toluene, reflux; (**v**) 3-Biphenyl-NH_2_, EDCI, HOBt·H_2_O, DMF, r.t.; (**vi**) cyclooctadiene iridium chloride dimer ([Ir(cod)Cl]_2_), bis(diphenylphosphino)methane (dppm), pinacolborane, CH_2_Cl_2_, r.t.; (**vii**) HCl, EtOAc, CHCl_3_, r.t. or R-COOH, EDCI, HOBt, DMF, r.t. or R-COCl, TEA, CH_2_Cl_2_, N,N-dimethyl-4-aminopyridine (DMAP), r.t.; (**viii**) NH_4_OAc, NaIO_4_, acetone, H_2_O, r.t., 48 h.

For the preparation of these compounds, it was first necessary to prepare the α-amino acid derivative (**57**), which was synthesized from diethyl aminomalonate (**56**). A series of transformations such as protection with Boc, hydrolysis, decarboxylation, and hydroboration using a cross-coupling catalyst, catalyst, led to the α-amino acid derivative [56]. After that, the main synthetic steps to obtain the desired compounds (Scheme 13) followed a process of deprotection of the amine, then condensation with an aryl carboxylic acid, and finally hydrolysis of the boronic ester (represented in molecule **58**) [56].

One problem associated with bortezomib is related to drug resistance and relapse in multiple myeloma. To overcome this, a very recent study [57] designed new dual targets that act on HDAC and on proteasome, based on other studies that suggest that overexpression HDAC might related to resistance of multiple myeloma to bortezomib. Through introduction of zinc-binding groups to peptide boronate with different amino acid residues, various compounds were obtained, being compound **66** the most promising. This compound **66** not only proved to inhibit both proteasome (IC_50_ of 1.1 nM) and HDAC (IC_50_ of 255 nM), but also showed antiproliferative activity against different multiple myeloma cell lines (RPMI-8226, IC_50_ of 6.66 nM; U266, IC_50_ of 4.31 nM; KM3, IC_50_ of 10.1 nM) including a cell line associated with multiple myeloma drug resistance to bortezomib (KM3/BTZ, IC_50_(**66**) of 8.98 nM; IC_50_(bortezomib) of 226 nM) [57].

**Scheme 14:**
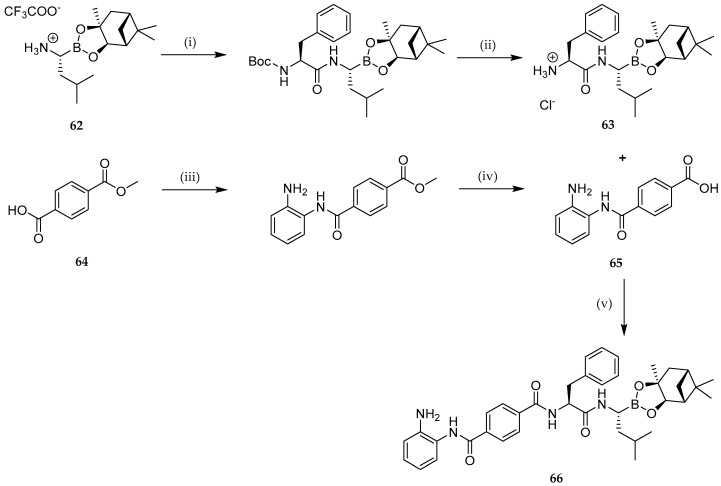
Synthesis of the HDAC and proteasome dual target inhibitor **66**. Reaction conditions: (**i**) (1) Boc-L-Phenylalanine, DMF, 0 °C; (2) TBTU, N-methylmorpholine (NMM), 16 h; (**ii**) HCl, EtOAc, 2 h; (**iii**) (1) Benzene-1,2-diamine, TBTU, DMF, 0 °C; (2) TEA, 8 h; (**iv**) LiOH, MeOH, 3 h; (**v**) (1) TBTU, DMF, 0 °C; (2) NMM, 8 h.

Compound **66** was synthetized (Scheme 14) through a two-step coupling reaction with 2-(1H-benzotriazole-1-yl)-1,1,3,3-tetramethylaminium tetrafluoroborate (TBTU). First, the intermediates **63** and **65** were synthetized separately: The intermediate **63** was obtained through a step of protection of **62** with boc-L-phenylalanine, followed by a process of deprotection; meanwhile, a coupling reaction of 4-(methoxycarbonyl)benzoic acid (**64**) with benzene-1,2-diamine was followed by a demethylation reaction, giving origin to intermediate **65**. The last step of the synthesis of compound **66** corresponds to a coupling reaction of **63** and **65** with TBTU [57].

### 2.2. Antibacterial Activity

Currently, antibiotic resistance is among the major concerns in human health. Bacteria resistance can usually be associated with the overexpression of different classes of β-lactamases by Gram negative bacteria, and the design of novel potential inhibitors of this enzyme is a very useful strategy. A well-known example of a β-lactamase inhibitor is the clavulanic acid [2,58,59,60,61]. The first report that boronic acids could act as β-lactamase inhibitors was made by a research group from Oxford University that noticed that borate ions inhibit β-lactamase I [62]. The capacity of boronic acids as inhibitors of β-lactamase is related with their ability to connect with the serine residue of this enzyme. The electrophilic boron group resembles the carbonyl present in β-lactam ring, and the replacement of the carbonyl with the boron leads to the formation of a tetrahedral adduct with serine that consequently inhibits these enzymes in a reversible way. The formation of this complex leads to the inhibition of the synthesis of bacteria cell wall [1,2,7,58,59,63,64]. The boronic acids that form this tetrahedral adduct and cause this inhibition can also be referred as boronic acid transition state inhibitors (BATSI), and are non-lactam transition state analogues. Specific interactions with various β-lactamases can be reached through introduction of different chemical groups on the carbon that carries the boronic acid group, providing an inhibitory activity in a precise manner for the different types of these enzymes. Because of these characteristics, studies about selective and potent boronic acid compounds are being made to discover novel compounds that can be administrated in combination with a β-lactam antibiotic agent [1,58,60,64,65].

**Scheme 15:**
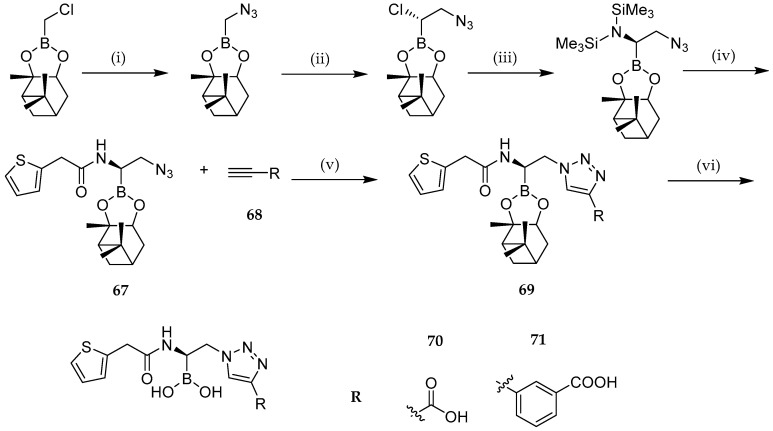
Synthesis of the α-amido-β-triazolylethaneboronic acids **70** and **71**. Reaction conditions: (**i**) NaN_3_, H_2_O, EtOAc, tetrabutylammonium iodide (TBAI); (**ii**) LiCHCl_2_, −100 °C; (**iii**) LiN(SiMe_3_)_2_, THF, −100 °C to r.t., overnight; (**iv**) (1) MeOH, THF, −10 °C to r.t., 1 h; (2) RCOCl, −10 °C, 1 h; (**v**) H_2_O, *tert*-BuOH, CuSO_4_, 60 °C, 18 h; (**vi**) MeCN, HCl, *n*-hexane, r.t., 3 h.

A very recent drug, vaborbactam, was approved by FDA in 2017 and by EMA in 2018, acting as a β-lactamase inhibitor [17]. Besides vaborbactam, other boronic acid compounds have shown the same activity. Some of these β-lactamase inhibitor boronic acid derivatives are chiral α-amido-β-triazolylethaneboronic acids **70** and **71** (Scheme 15). The design of these compounds was based on the knowledge that boronic acid is a bioisostere of the carbonyl present in β-lactam [58]. This knowledge also lead the creating of a BATSI with higher affinity. These compounds bind covalently and reversibly to serine residue S318, tyrosine residue Y150, and lysine residue K67 of class C β-lactamases, being the hydroxyl group of boronic acid responsible for this inhibitor-enzyme interaction. The triazole substituent was also used as a bioisostere of phenyl in order to facilitate the synthesis process and cell penetration [58]. It was proved that compounds **70** and **71** were very effective against problematic highly resistant bacteria strains found in hospitals (**70**, inhibitory constant (K*i*) of 0.004 µM; **71**, K*i* of 0.008 µM) [58].

The initial step of the stereoselective synthesis of the compounds **70** and **71** (Scheme 15) corresponds to the production of α-acylamido-β-azido derivative **67**, followed by a reaction with alkynes **68** and, at last, the deprotection of the boronic ester moiety (represented in molecule **69**) occurs by transesterification with phenylboronic acid. The formation of the triazole is regioselective, and the desired product is obtained at quantitative yields [58].

**Figure 7:**
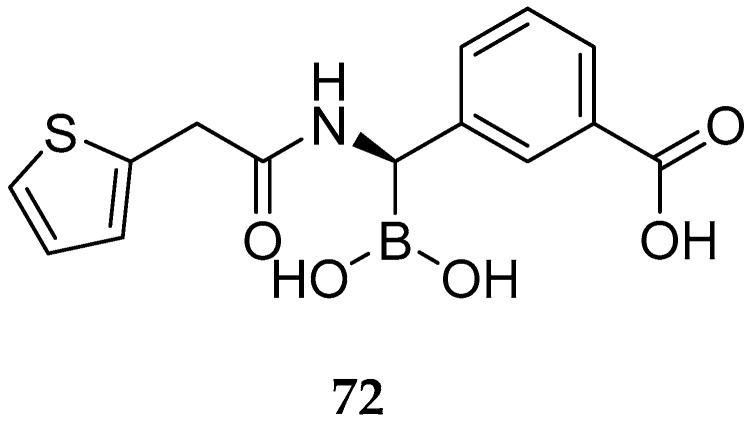
Compound SM23 (**72**), an inhibitor of class C β-lactamases and of biofilms formation.

Another studied boronic acid β-lactamase inhibitor was compound SM23 (**72**, Figure 7). This compound is also a BATSI and is considered as one of the best inhibitors of class C β-lactamases reported to date. Its design was based on the mimetic approach, in which the different chemical groups with a suitable stereochemistry were arranged with a boronic acid in order to resemble the natural substrate β-lactam [60,64]. SAR studies revealed that the introduction of a group with negative charge in the side chain, such as a carboxylate, improves binding affinity and cell penetration and that one of the hydroxyl groups of boronic acid is important to form an interaction with the nitrogen of serine residues (Ser64 and Ser315) in the oxyanion hole of the active site of β-lactamases [64]. A recent publication revealed another interesting application of compound **72** as an inhibitor of the formation of biofilms by Pseudomonas aeruginosa, a Gram-negative bacteria that is responsible for many nosocomial infections in immunosuppressive patients and is very resistant. When exposed to SM23 (**72**), it is observed inhibition in the production of virulence factors related to the formation of biofilms of *P. aeruginosa*, like pyoverdine and elastase, without co-administration of an antibiotic [60].

The synthesis of **72** was accomplished using the same approach of compounds **70** and **71** (Scheme 15).

To obtain a β-lactamase inhibitor that could act on a larger spectrum and also to achieve oral administration, a cyclic boronic acid (**75**) was synthetized. Biological activity studies showed that compound **75** has high inhibitory activity against the β-lactamases serine enzyme of classes A, C, and D, and also against the metalloenzymes [66]. The fact that one single compound can have potent activity against serine β-lactamases and metalloenzyme is very attractive (minimum inhibitory concentrations (MICs) of compound **75** when combined with commercially available β-lactam antibiotics are represented in Table 5). In serine enzymes, the covalent binding of the boron with the catalytic serine residue is done in a “slow-binding” kinetics, while in the metalloenzymes covalent bond occurs between the boron and the water molecule of the enzyme and in a “fast-on, fast-off” type of kinetics. Other interesting characteristics that contribute for the attention given to this compound **75** are revealed by in vivo studies in rats, showing similar pharmacokinetic properties to β-lactam antibiotics and a good oral bioavailability, which allows it not only to be administrated intravenously but also orally. Besides, no toxic effects were shown [66].

**Scheme 16:**
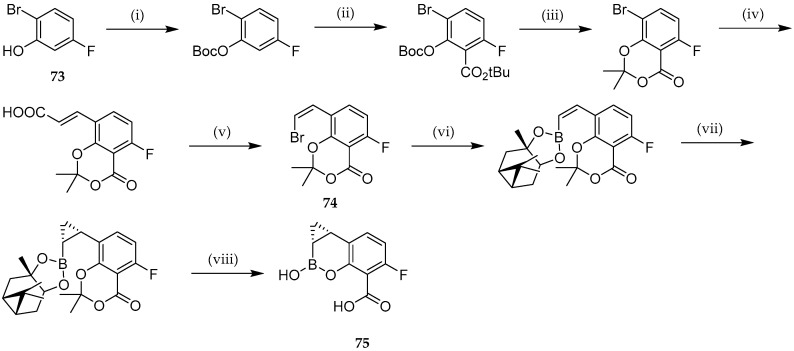
Synthesis of the cyclic boronic acid **75**, inhibitor of serine β-lactamases and metallo enzymes. Reaction conditions: (**i**) Boc_2_O, DMAP, CH_2_Cl_2_, r.t., 30 min; (**ii**) (1) Lithium diisopropylamide (LDA), THF, −78 °C, 1 h; (2) Boc_2_O, DMAP, CH_2_Cl_2_, r.t., overnight; (**iii**) (1) TFA, CH_2_Cl_2_, r.t., 16 h; (2) acetone, TFA, trifluoroacetic anhydride (TFAA), 70 °C, overnight; (**iv**) acrylic acid, Pd(OAc)_2_, tris(o-tolyl)phosphine (P(o-toly)_3_), TEA, DMF, 100 °C, 14 h; (**v**) (1) Br_2_, CHCl_3_, 0 °C, 2 h; (2) TEA, DMF, 0 °C to r.t., 8 h; (**vi**) B_2_((+)pinanediol)_2_, PdCl_2_(dppf), KOAc, dioxane, 60 °C, 2 h; (**vii**) CH_2_N_2_, Pd(OAc)_2_, THF, −20 °C to r.t., 12 h; (**viii**) (1) column chromatography; (2) 3 N NaOH, dioxane, r.t., 30 min; (3) TFA, triethylsilane (TES), i-BuB(OH)_2_, 0 °C to r.t., 30min.

To achieve the synthesis of compound **75** (Scheme 16), the key steps that needed to be accomplished were: Boc protection of phenol **73**, deprotonation, then acylation of the aromatic ring, Heck reaction, bromination (represented in molecule **74**), borylation catalyzed by palladium, palladium-catalyzed cyclopropanation, and finally hydrolysis of protecting groups [66].

In addition to overexpression of β-lactamases in bacteria, another known resistance mechanism is related to efflux pumps, thus the discovery and design of novel antibacterial drugs that could act by inhibiting the action of these pumps is an exciting approach [67,68]. It has been recognized that boronic acid derivatives can be promising inhibitors of chromosomal NorA efflux pump of *Staphylococcus aureus*. These pumps are involved in bacterial resistance to the antibacterial fluoroquinolones, specially to hydrophobic fluoroquinolones such as ciprofloxacin [67]. Synthetic inhibitors of efflux pumps are usually based on heterocyclic compounds and in pump efflux inhibition assays often use the alkaloid reserpine as a positive control, but this alkaloid is not used in therapy due to neurotoxicity at therapeutic concentrations (minimum modulatory concentration (MMC) of 4–8 µg/mL). Due to the formation of a stable transition complexes with sugars and amino acids, heterocyclic boronic acid compounds were tested for the inhibition of NorA efflux pump, and compounds 6-(3-phenylpropoxy)pyridine-3-boronic acid (**76**) and 6-(4-phenylbutoxy)pyridine-3-boronic acid (**77**) proved to be efficient. A SAR study proved that—(i) the best results were obtained when boronic acid is at the para position; (ii) boron atom is essential, since the replacement by carbonylated and hydroxymethyl analogues did not show any activity; (iii) esterification of the boronic acid leads to a decrease in activity; (iv) an aryl in C-6 position retains inhibitory activity. It was also shown that these compounds (**76** and **77**) have no intrinsic antibacterial activity and can be used to potentiate the activity of the quinolone antibiotics ciprofloxacin and norfloxacin (Table 6), promoting the accumulation of ethidium bromide (a NorA pump fluorescent substrate), and showing no undesirable cytotoxicity due to the selectivity for bacterial efflux pumps (no effect on human efflux pump P-glycoprotein) [67].

**Scheme 17:**
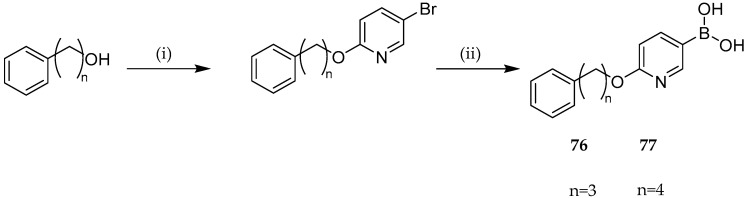
Synthesis of NorA efflux pump boronic acid inhibitors compounds **76** and **77**. Reaction conditions: (**i**) (1) NaH, anhydrous THF, 0 °C to r.t., 30 min then reflux, 1 h; (2) 5-bromo-2-fluoropyridine, reflux, 12 h; (**ii**) (1) n-BuLi, anhydrous ether, −78 °C, 1 h; (2) B(OiPr)_3_, −78 °C to r.t., 1 h; (3) 3 N HCl.

The synthetic process of the compounds **76** and **77** (Scheme 17) was based on the halogen–lithium exchange reaction with subsequent boronation [67].

**Scheme 18:**
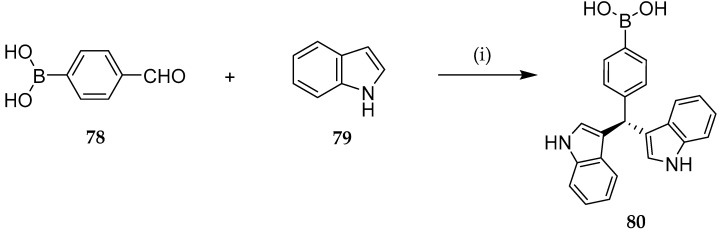
Synthesis of the bis(indolyl)methane boronic acid derivative **80**, a leucyl-tRNA synthetase inhibitor. Reaction conditions: (**i**) Fe(ox)–Fe_3_O_4_, H_2_O, reflux.

Another interesting boronic acid derivative that proved to have antibacterial activity is the bis(indolyl)methane boronic acid derivative **80**. This compound was discovered in a study with the purpose of testing a series of bis(indolyl)methane compounds that could be used for the treatment of methicillin-resistant *Staphylococcus aureus* (MRSA), a type of infection that often occurs in hospitals, and that is difficult to treat. In that study [69], a set of bis(indolyl)methane derivatives were synthetized (Scheme 18) where only the substituent attached to the aryl or indole moiety differed. It was discovered that when boronic acid was attached to the aryl moiety, the activity against MRSA was observed (MIC of 7.81 µg/mL), and no toxicity was revealed. Docking analysis also predicted that the action of bis(indolyl)methane boronic acid derivative could be related to binding to leucyl-tRNA synthetase, an enzyme that plays an important role in bacterial protein synthesis. It was also revealed that the action of this derivative **80** might be bactericidal and that it is more specific against Gram positive bacteria, which might be related to the peptidoglycan layer [69].

The synthesis of compound **80** was mediated through a Fe(ox)–Fe_3_O_4_ promoted condensation reaction, reacting aldehyde with a boronic acid (**78**) substituent with indoles (**79**) in water [69].

### 2.3. Antiviral Activity

The capacity of boronic acids to bind to carbohydrates allows their use for the treatment the virus with high glycosylated envelopes, such as the human immunodeficiency virus (HIV) [7]. Once again, their physicochemical characteristics proved to be associated with this biological application. 

**Figure 8:**
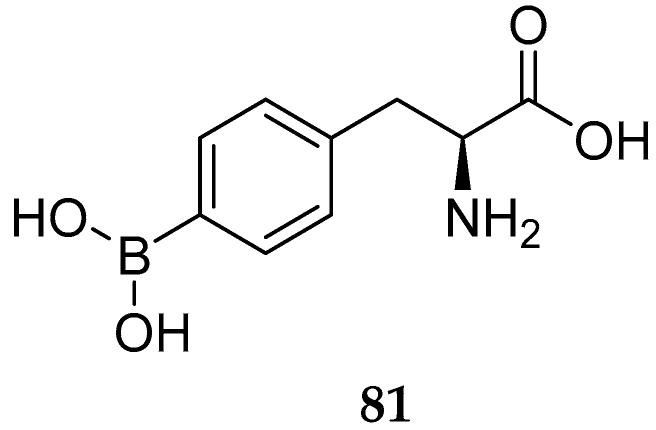
BPBA compound (**81**), disruptor of Rev-RRE interaction and inhibitor of HIV-1 replication.

One of the diseases considered a universal health problem is the acquired immune deficiency syndrome (AIDS), and it is caused by the human immunodeficiency virus type 1 (HIV-1) [70]. The Rev response element (RRE) is an RNA sequence of HIV-1 with an important role in the proliferation of this virus. Through interaction with Rev protein, RRE allows virus RNA replication contributing to its life cycle. Therefore, this Rev-RRE interaction is considered as a promising drug target for HIV treatment [70,71]. Branched peptides (BPs) are a good targeting strategy, since they form strong interactions with the transactivation response element (TAR) of HIV, that is related to the virus RNA, and also proved to have no cytotoxicity associated, good cell permeability, and can bind to TAR at submicromolar levels. Based on the fact that boronic acids form reversible covalent bonds with Lewis bases and that this kind of bases are present in RNA of HIV, for instance 2′-and 3′-hydroxyl groups, boronic acid moiety was introduced to these BPs [70]. Thus, this introduction could enhance the affinity of branched peptide boronic acids (BPBAs) towards RNA. A series of BPBAs were synthesized through split and pool synthesis, and it was proved that these compounds increase the affinity towards RRE and, by binding to this RNA sequence, the interaction Rev-RRE is disrupted, consequently leading to inhibition of viral replication [70,71]. SAR studies revealed that when boronic acid moiety was removed, a decrease in affinity was shown and also that the acidity of boron is important for binding affinity, since the introduction of an electron-withdrawing group placed in ortho the boronic acid increased the Lewis acidity of boron and subsequently, their binding affinity [70]. An increase in acidity of boronic acids is associated with the increase of electrophilicity and thus can form more stable complexes [5,70]. The best compound was BPBA3 (**81**, Figure 8), which showed a great cellular uptake and inhibition of p24 capsid protein of HIV and HIV replication in vitro (IC_50_ of ≈ 5 µM). The binding of this compound to RRE suggests a conformational change of the tertiary structure of the RNA, which might be more predisposed to be cleaved by RNases, the enzymes responsible for defense mechanism to RNA virus [71].

Another strategy for HIV treatment is through the design of HIV-1 protease inhibitors. The HIV-1 protease is also critical in viral replication, so inhibitors of this enzyme are a good approach for developing HIV antiviral drugs. The drug darunavir is known to be a HIV-1 protease inhibitor and its mechanism of action consists in targeting the S2′ enzymatic subsite, forming hydrogen bonds with the amides present in Asp30 [72,73]. An ideal protease inhibitor must have the ability to interact with three local sides; these include the main chain and side chain of Asp30 and a molecule of water coordinated to the main chain of Gly48. Based on the knowledge that boronic acids can form strong hydrogen bonds and these bonds are essential for this inhibitory activity, a 4-sulfonylphenylboronic acid (compound **84**) was designed through replacement of the 4-sulfonylaniline of darunavir, and the effects of this substitution were analyzed [72]. It was observed that the boronic acid **84** acts as a competitive inhibitor of HIV-1 protease and that the affinity towards the target was 20-fold greater than darunavir (darunavir, K*i* of 10 pM; boronic acid derivative **84**, K*i* of 0.5 pM). X-Ray crystallography studies proved that the boronic acid moiety interacts with all three locals referenced above, thus contributing to a significantly better affinity. Besides these proprieties, this boronic acid (**84**) is non-toxic to human cells and, unlike aniline moiety in darunavir, has no related genotoxicity as a result of in vivo metabolic activation, since that the product of metabolic phase I activation in boronic acids is an alcohol, later metabolized in phase II in order to be eliminated from the body [72].

**Scheme 19:**
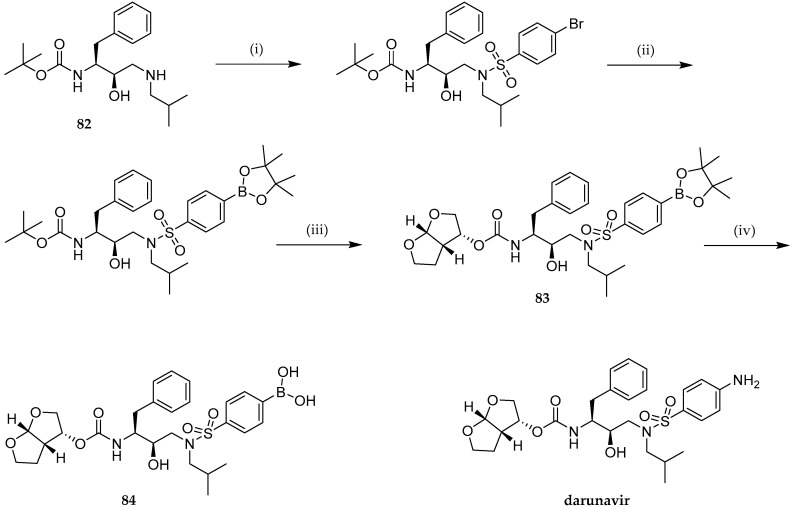
Synthesis of HIV-1 protease inhibitor **84** based on the drug darunavir. Reaction conditions: (**i**) TEA, 4-bromobenzenesulfonyl chloride, CH_2_Cl_2_, dry N_2_ atmosphere, 0 °C, 16 h; (**ii**) KOAc, B_2_pin_2_, Pd(dppf)Cl_2_–CH_2_Cl_2_, 1,4-dioxane, 80 °C, 24 h; (**iii**) (1) HCl, dioxane, r.t., 4 h; (2) TEA, 2,5-dioxopyrrolidin-1-yl ((3R,3S,6R)-hexahydrofuro[2,3b]furan-3-yl) carbonate, r.t., 16 h; (**iv**) acetone/H_2_O, NaIO_4_, NH_4_OAc, r.t., 12 h.

The synthesis of **84** (Scheme 19) was accomplished by a palladium-catalyzed coupling reaction. The initial step corresponds to a coupling reaction between the starting material **82** and 4-bromobenzenesulfonyl chloride, followed by a palladium-coupling reaction with B_2_pin_2_ and further addition of 2,5-dioxopyrrolidin-1-yl((3R,3S,6R)-hexahydrofuro[2,3b]furan-3-yl) carbonate. The hydrolysis of intermediate **83** leads to the final product **84** [72].

The specific binding between influenza A virus (IAV) glycoprotein hemagglutinin (HA) and the glycan receptor of the host is crucial for the infection and transmission of this virus in humans. This virus uses as receptors for endocytosis the glycans that are present in cell surface and also possesses glycan residues to interact with host cells. Since boronic acids can form stable complexes with saccharides, compounds in which boronic acids were added to quinolones, were designed (**87**–**92**) with the purpose of disrupting the binding of the virus to glycans in host cells [74]. The boronic acid moiety was introduced in quinolones because these derivatives are known to interact with DNA and also to have anti-viral activities [74,75]. This study suggests that the boronic acid is important to interfere with the importation of ribonucleoprotein (RNP) of IAV, complexes that are important for DNA replication of this virus, and delay the virus access into the nucleus of host cells. These compounds, especially compound **88**, proved to inhibit mitogen-activated protein kinase (MAPK) and nuclear factor-kappa B (NF-κB) signalling pathways—it is thought that MAPK and NF-κB can be involved in IAV replication and viral RNA (vRNA) synthesis, respectively. SAR studies demonstrated that the boronic acid is critical for anti-IAV activity of these quinolone derivatives and, moreover, it enhances the anti-IAV activity, both in vitro and in vivo assays (in vitro: **88**, IC_50_ of 2.5 µM; oseltamivir, IC_50_ of 9.5 µM). Another interesting fact is that compound **88** also revealed an inhibitory activity in other virus strains. Moreover, rats treated with this compound displayed a higher survival rate for IAV than the rats treated with the commercially available drug oseltamivir [74].

**Scheme 20:**
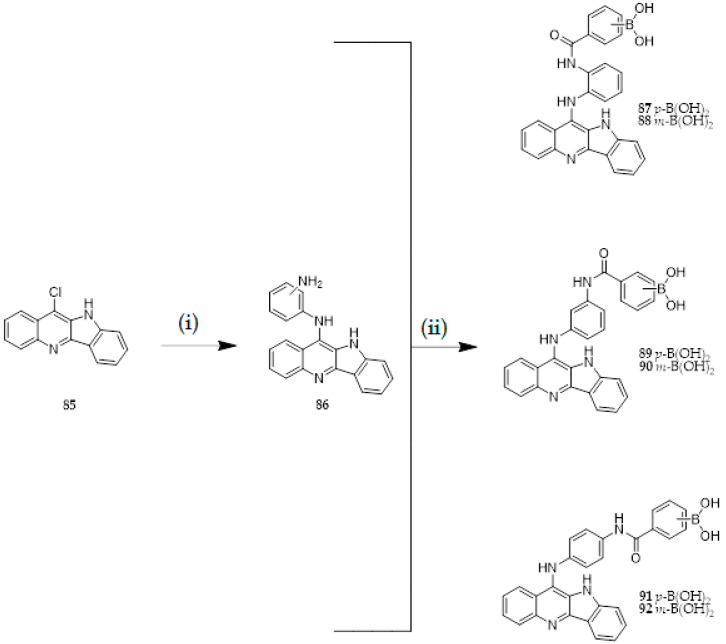
Synthesis of boronic acid derivatives **87**–**92** as IAV replication inhibitors. Reaction conditions: (**i**) Aromatic diamine, HCl, 2-ethoxyethanol, 100 °C, 2 h; (**ii**) 4-carboxyphenylboronic acid or 3-carboxyphenylboronic acid, 4-(4,6-dimethoxy-1,3,5-triazin-2-yl)-4-methyl-morpholinium chloride (DMT-MM), 2-ethoxyethanol, r.t., 18 h.

For the synthesis of these compounds (Scheme 20), the first step involved a substitution reaction of 11-chloroquindoline derivative **85** with an aromatic diamine (represented in molecule **86**), followed by amidation with 3- or 4-carboxyphenylboronic acid [74].

**Figure 9:**
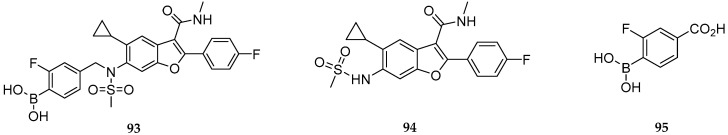
NS5B polymerase inhibitor for HCV treatment (**93**) and its metabolites (**94** and **95**).

Hepatitis C virus (HCV) is responsible for chronic HCV infection, which affects millions of people worldwide, leading to liver cirrhosis and hepatocellular carcinoma. The design of already existing class of antiviral compounds used for HCV, the non-structural protein 5B (NS5B) polymerase inhibitors, is complicated due to lack of homology with the binding sites of different genotypes of HCV and the quick rise of mutants that are resistant to HCV drug [76]. The study of the introduction of boronic acids in NS5B inhibitors was done to investigate if it could bring advantages for targeting HCV that are resistant to NS35. Boronic acids are known for reversible covalent inhibition of hydroxyl proteases, and thus the study of boronic acid compounds as HCV protease inhibitors was accomplished [22,76]. In the case of mutants resistant to NS5B polymerase inhibitors, the introduction of boronic acid group to this class of antivirals (**93**, Figure 9) revealed to be essential for the inhibition of HCV RNA-dependent RNA polymerase (**93**, half maximal effective concentration (EC_50_) of 3.2 nM). This phenomenon occurs in the initial step of RNA polymerase replication cycle. It is thought that this happens through stabilization of β-flap, a portion of NS5B that is important in the regulation of polymerase initiation and binding of template RNA, in an inactivated state [76].

Compound **93** entered in clinical trials but, unfortunately, the formation of two metabolites (compounds **94** and **95**, Figure 9) through an easy benzylic oxidation with significantly higher half-life times (t_1/2_(**94**) = 5 h; t_1/2_(**95**) = 45 h) led to the discontinuation of this clinical trial (present in Table 1) [77,78]. The low half-life time value in the parent compound (**93**) implies a high daily dose of this drug for efficacy [78]. With the goal of improving the pharmacokinetic proprieties to avoid the formation of these metabolites and to maintain the efficacy of this compound, a second generation of NS5B inhibitors were designed by the same research group, in which a N-phenyl derivative (**100**) revealed to be the most promising one [78]. To obtain this N-phenyl benzoxaborole **100**, the used strategy was based on the elimination of the benzylic carbon in compound **93**. Pharmacokinetic studies showed that this derivative could be a good candidate for clinical trials, since: (i) Comparing to compound **93**, clearance improved in mice, rats, dogs and monkeys—lower clearance can correlate with the elimination of benzylic oxidation; (ii) no inhibition of cytochrome P450 (CYP) isoform 2C9 was observed—inhibition of this enzyme could lead to safety problems; (iii) no formation of the metabolite **94** either in vitro or in vivo [78]. It is thought that, instead of benzylic oxidation, the benzoxabolore goes through oxidative-deborylation [78].

**Scheme 21:**
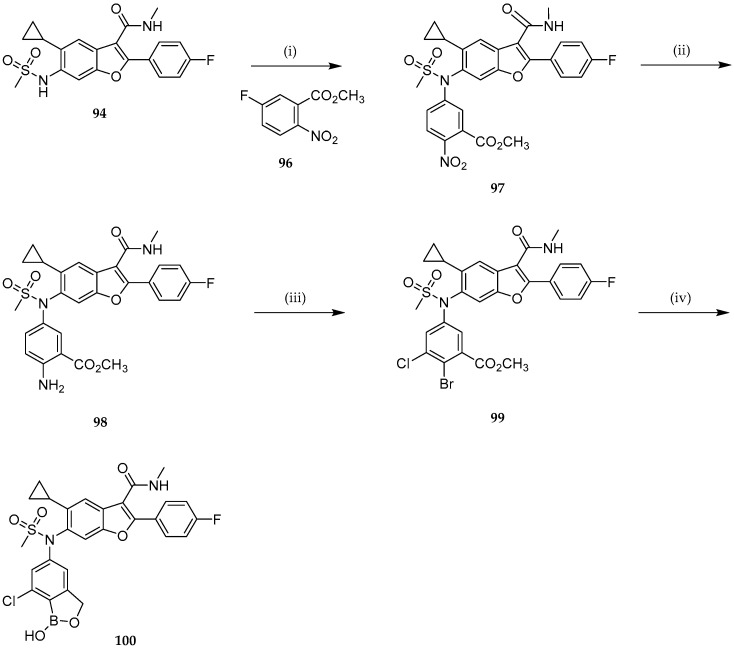
Synthesis of NS5B inhibitor second generation derivative **100**. Reaction conditions: (**i**) Methyl 5-fluoro-2-nitrobenzoate, Na_2_CO_3_, DMF, 70 °C, 72 h, (**ii**) palladium on carbon (Pd/C), THF/EtOH,, H_2_, r.t., 24 h; (**iii**) (1) DMF, 60 °C, 5 min; (2) MeCN, HBr, NaNO_2_, H_2_O, 0 °C, 30 min; (3) CuBr, 50 °C, 30 min; (4) MeOH, LiBH_4_/THF, −5 °C, 2 h; (5) THF, DIPEA, chloromethyl methyl ether (MOM-Cl), 50 °C, 18 h; (**iv**) (1) B_2_Pin_2_, Pd(dppf)Cl_2_, 1,4-dioxane, N_2_, 108 °C, 22 h; (2) THF, MeOH, HCl, 70 °C, 18 h.

The synthesis of this second generation NS5B inhibitor (**100**, Scheme 21) was done through nucleophilic aromatic substitution (S_N_Ar) coupling of metabolite **94** with the nitro fluorobenzene substrate (**96**). The nitro group of the intermediate **97** suffered a reduction forming aniline **98**. This reaction was followed by a process of chlorination ortho-to aniline group and subsquent Sandmeyer reaction to change the NH_2_ of the aniline to a bromine. Compound **99** was produced through a reduction reaction with further protection with chloromethyl methyl ether (MOM-Cl). Finally, compound **100** was synthetized through an acid-cleavage reaction [78].

Flavivirus is responsible for several types of infections like Zika, West Nile, and Dengue virus infections, being the inhibition of flaviviral proteases an interesting approach for the treatment of these infections, particularly, with enzyme inhibitors that could act by covalent binding. Therefore, a research group analyzed the effect of the boronic acid moiety in the inhibition of flaviviral proteases, and a series of dipeptide boronic acids (exemplified herein with compound **105**) were synthetized [79]. A SAR study showed that the activity is related to boronic acids present at the C-terminus and that the replacement of boronic acids with amides led to almost inactive compounds. The presence of boronic acids led to a 1000-fold higher affinity towards flaviviral proteases (K*i* values of **105** in Table 7) and no cytotoxicity was observed. It was also proved that the boronic acid **105** forms a hydrogen bond with the oxygen of the active site of Ser135 residue of the flaviviral protease [79].

**Scheme 22:**
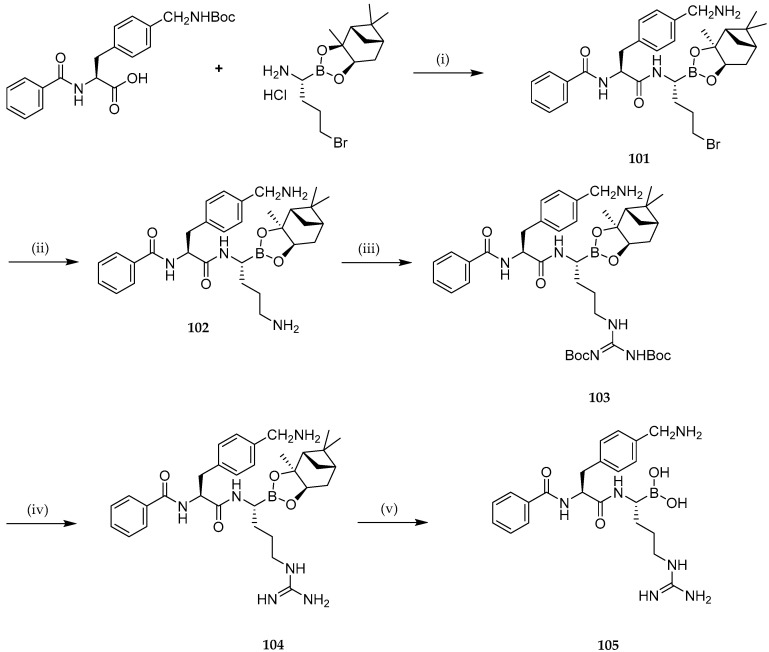
Synthesis of the flaviviral protease inhibitor **105** with boronic acid group. Reaction conditions: (**i**) (1) NMM, isobutyl chloroformate (IBCF); (2) (R)-4-bromo-1-((3aS,4S,6S,7aR)-3a,5,5-trimethylhexahydro-4,6-methanobenzo[d][1,3,2]dioxaborol-2-yl)butan-1-amine, CH_2_Cl_2_, DIPEA, THF, −15 °C to r.t., overnight; (**ii**) (1) NaN_3_, DMF, 100 °C, 1 h; (2) H_2_, Pd/C, MeOH, r.t., overnight; (**iii**) bis-Boc-pyrazole-1-carboxamidine, DMAP, MeOH, r.t., 48 h; (**iv**) TFA, CH_2_Cl_2_, HCl, r.t., overnight; (**v**) PhB(OH)_2_, H_2_O, diethyl ether, HCl (aq.), r.t., overnight.

The approach to synthesize compound **105** (Scheme 22) starts with a reaction with N-methylmorpholine (NMM), with subsequent coupling reaction, leading to the formation of bromide intermediate **101**. This intermediate was then transformed into azide **102** and, after hydrogenation, converted into Boc-protected guanine **103**. The guanidine group formed was then deprotected. The final step corresponds to the deprotection of the boronic ester **104** using TFA [79].

### 2.4. Sensors and Delivery Systems

**Figure 10:**
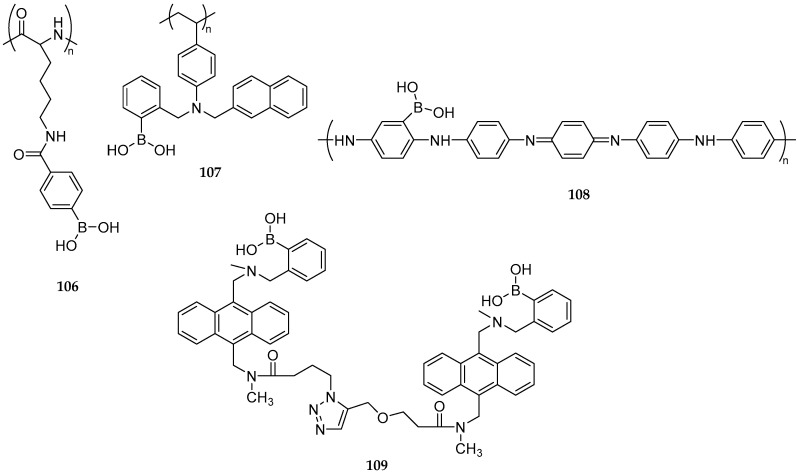
Boronic acid derivatives as sensors. **106**: Poly(L- and D-lysine) with phenylboronic acid as a glucose sensor; **107**: Boronic acid derivative with a fluorophore for saccharide sensing; **108**: Poly(aniline boronic acid) polymer as a dopamine sensor; **109**: Anthracene-based diboronic acid, an oligosaccharide sensor.

Although most of the mentioned biological applications of boronic acid derivatives are related with their role as enzyme inhibitors, some boronic acid derivatives can be used as sensors and in drug delivery systems. The ability of boronic acids to react reversibly and covalently with 1,2- and 1,3- diols allows them to be sensor of carbohydrates, such as glucose, and therefore they can be useful for monitoring diseases such as diabetes [6,8]. This sensing is possible because upon binding with saccharides, a change is absorption is observed. Changes in absorption spectra can be related to steric effects due to the saccharide interaction with boronic acids or it can also be related to hydrogen bond loss that occurs when boronic acids are converted to boronic esters upon interaction with glucose. These changes in absorption can be measured by complexation between a boronic acid with a dye. One example is a poly(L- and D-lysine) modified with phenylboronic acid (**106**, Figure 10), considered as optical sensor [6,10,80].

There are also some fluorescence quenching-based saccharides sensors [6,10,80,81]. Examples of this kind of sensors are photoinduced electron transfer (PET) materials with boronic acid. Polymers with boronic acids and a fluorophore, for example, an amine (**107**, Figure 10), in the presence of saccharides produce increased fluorescence, having a higher specificity for glucose. The interaction between this polymer and a saccharide decreases PET, leading increased fluorescence emission [6,10,80]. Although promising, this polymer presents some disadvantages: Poor water solubility, interference with external factors such as the solvent, poor stability, and is easily decomposed by light [80].

Besides sensing saccharides, boronic acid polymers can also be used in patients with diabetes for insulin delivery. The polymers used are mainly hydrogels that disintegrate or swell upon hydrophilicity increase in glucose presence, allowing the release of insulin [6]. Poly(aniline boronic acid) polymers (**108**, Figure 10) can be used not only for saccharide sensing but also in dopamine sensing [6,10,80]. Variations in dopamine concentration are associated with some neurodegenerative diseases such as Parkinson’s disease, and the detection of the concentration of this neurotransmitter can be measured by the oxidation of dopamine to dopamine-o-quinone with electrodes. The use of boronic acids in this method provides a high selectivity of electrodes and therefore a more precise detection [6].

Recently, a sensor with the ability to recognize Lewis oligosaccharides selectively was developed and could be useful in the early detection and diagnosis of cancer. This sensor is an anthracene-based diboronic acid and it can recognize HEPG2 cells (**109**, Figure 10) [80].

## 3. Biological Applications of Other Boron Containing Compounds

Besides boronic acids, there are other boron containing compounds with interesting and promising therapeutic applications. This section will briefly discuss the application of boron compounds, such as diazaborines, benzoxaboroles, and boronic esters.

**Figure 11:**
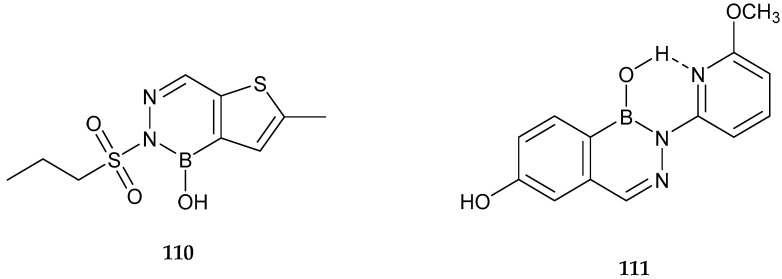
Examples of bioactive diazaborines. **110**, compound with antibacterial activity; **111**: Compound with anticancer activity.

Diazaborines were one of the first compounds with boron atom that were investigated for their therapeutic proprieties. Diazaborines were identified as antimicrobial agents by Gronowitz et al. that observed that these compounds share some similarities with the antibiotic nitrofurantoin [82,83]. These compounds are mainly effective against Gram-negative bacteria like *Escherichia coli*; for instance, compound 110 (Figure 11) is potent against this bacterium (MIC of 1.25 μg/mL) [84].

In addition to this activity, diazaborines also proved to be effective against breast cancer. Compound **111** (Figure 11) mimics estradiol structure and proved to have an antiproliferative activity against human breast cancer cell line MCF-7, with an approximate IC_50_ value of 5 μmol/L [85].

Other well-known compounds containing boron and with interesting bioactivity are benzoxaboroles. These compounds are characterized by incorporating boron atom in a heterocycle that is attached to an aromatic ring [80]. Since the discovery of the antifungal benzoxaborole drug tavaborole (compound **112**), the interest of these compounds in Medicinal Chemistry has been increasing [86,87,88]. In comparison to boronic acids, they are usually more soluble compounds [89].

They are mainly known for their application as anti-infective and antiparasitic activities. In Table 8, it is summarized some of the most recent reported activities of benzoxaborole compounds.

**Figure 12:**
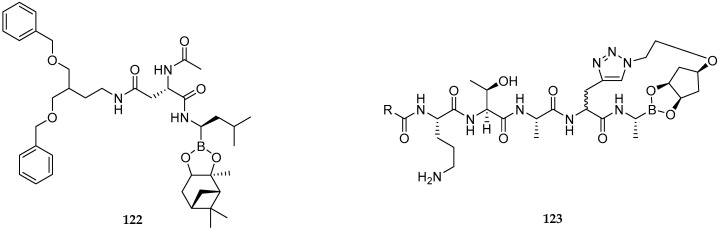
Boronic esters as promising compounds in Medicinal Chemistry. **122**: Selective proteasome inhibitor and apoptotic agent; **123**: Antibacterial agent against *E. coli*, *P. aeruginosa*, *K. pneumoniae*, and *S. aureus*.

Besides boronic acids, some boronic esters also seem to be promising compounds in Medicinal Chemistry, especially as anticancer (exemplified with 122) and antibacterial agents (exemplified with 123). Compound **122** (Figure 12) was discovered as a selective proteasome inhibitor (ChT-L, IC_50_ of 2.7 nM; HCT116, IC_50_ of 31.8 nM), acting as a growth inhibitor through induction of G2/M cell cycle arrest and, through p53 accumulation, and proved to also act as a pro-apoptotic compound [100]. As antibacterial agents, compound 123 (Figure 12) proved to be effective against *E. coli* type I signal peptidase (EcLepB), an enzyme that produces a vast number of crucial proteins for survival virulence of this bacteria. Besides inhibiting this enzyme (MIC of 4–16 μg/mL), this molecule demonstrated effects on bacterial type I signal peptidase (SPase I) in other bacteria strains, such as *P. aeruginosa* (MIC of 32–64 μg/mL), *Klebsiella pneumoniae* (MIC of 8 μg/mL), and *S. aureus* (MIC of 2 μg/mL) [101].

## 4. Conclusions

**Figure 13:**
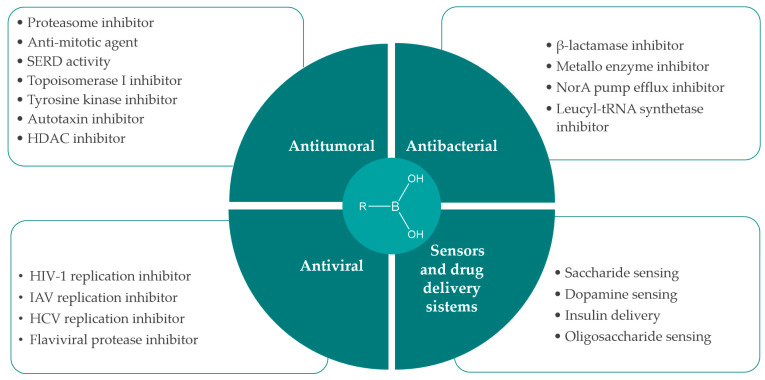
Biological applications and respective activities of boronic acid derivatives.

Boron compounds in Medicinal Chemistry have been neglected until the discovery of bortezomib, a peptide boronic acid proteasome inhibitor drug with anticancer activity. After this drug, other boronic acid derivative drugs have been developed, for instance, ixazomib and vaborbactam. The lack of interest in these compounds was mainly due to the stigma that boron atom was associated with toxicity, which has been proven to be a false concept. Due to the chemical characteristics of boronic acids and the subsequent ability to form reversible tetrahedral bonds with substrates such as enzymes, saccharides, and nucleic acids, the use of this moiety in several compounds for therapeutic application has been arising. Throughout this review, it has been shown the diverse applications of boronic acids in therapy, being the most relevant as anticancer, antibacterial, and antiviral agents and as sensors or delivery systems, as resumed in Figure 13.

From a Medicinal Chemist perspective, the following considerations can be taken into account: (i) Introduction of boronic acids can improve various proprieties, including pharmacokinetic characteristics, such as bioavailability and toxicity; (ii) all of these proprieties are due to the physicochemical characteristics of boronic acids; (iii) the synthetic approaches to obtain these compounds are well known and relatively easy to accomplish; (iv) although boronic acid is the most common studied group, other boron containing molecules also proved to have interesting applications. It is therefore important to continue the investigation of the capacities of boron containing compounds in Medicinal Chemistry, in order to improve already known compounds or to obtain new ones.

## Figures and Tables

**Table 1:** Some of the completed and ongoing clinical trials with boronic acid compounds and other boron-containing compounds. Source: www.clinicaltrials.gov (accessed on May 2020).

Study	Purpose	Phase	Status
Combination therapy with bortezomib in advanced stage aggressive lymphomas	To study whether the combination of CHOP ^1^ with bortezomib to overcome drug resistance induced by pro-apoptotic proteins BAX and BCL-XS ^2^	Phase 1 Phase 2	Completed (October 2009)
Combination therapy with ixazomib for the treatment of sarcoma	To establish a safe and tolerable dose combination of selinexor and ixazomib when used together for the treatment of certain types of advanced sarcoma	Phase 1	Ongoing
Lymphoma study in sombination with bortezomib or vorinostat	To determine the safety, tolerability and MTD ^3^ of AMG-655 ^4^ when combined with bortezomib or vorinostat	Phase 1	Completed (August 2011)
Bortezomib in relapsed or refractory AIDS ^5^-related sarcoma	To evaluate the MTD of bortezomib in patients with AIDS ^5^-related Kaposi sarcoma	Phase 1	Completed (January 2015)
Combination therapy with bortezomib in ovarian epithelial cancer, fallopian tube cancer, or primary peritoneal cancer	To determine the MTD and dose-limiting toxicities of intraperitoneal bortezomib when combined with intraperitoneal carboplatin	Phase 1	Completed (January 2018)
Combination therapy with bortezomib in acute myeloid leukemia	To identify the effective and tolerable dose of the bortezomib/sorafenib combination in acute myeloid leukemia; to recommend a dose level for phase 2 and define specific toxicities	Phase 1	Ongoing
Combination therapy with bortezomib in lymphoma	To determine the RD ^6^ for phase 2 dose of alisertib and bortezomib when combined with rituximab in patients with relapsed/refractory mantle cell and B-cell low grade non-Hodgkin lymphoma	Phase 1	Ongoing
First time in human safety and pharmacokinetics study of GSK3036656 in healthy subjects	To evaluate the safety, tolerability and pharmacokinetics of single ascending and repeat oral doses of GSK3036656 in healthy adults	Phase 1	Completed (August 2017)
Intravenous ixazomib in pediatric participants with relapsed or refractory acute lymphoblastic leukemia or lymphoblastic lymphoma	To determine the MTD and/or phase 2 RD, safety and toxicity, and pharmacokinetics	Phase 1	Ongoing
Ixazomib and pevonedistat in treating multiple myeloma that has come back or does not respond to treatment	To study the side effects and best dose of pevonedistat when combined with ixazomib in multiple myeloma that has come or does not respond to treatment	Phase 1	Ongoing
The effect of ixazomib on the latent HIV reservoir	To determine the safety and tolerability of ixazomib in HIV infected patients who are on ART ^7^ that suppresses HIV replication; to determine the effect of ixazomib on the size of the HIV reservoir	Phase 1	Ongoing
Dose escalation study of nelfinavir and ixazomib in patients with advanced solid tumors or lymphoma	To determine the MTD of the combination in advanced solid tumors or lymphoma	Phase 1	Ongoing
Safety, tolerability and pharmacokinetics of multiple rising doses of ixazomib in lupus nephritis	To characterize the safety and tolerability of ixazomib when administered as multiple oral doses at escalating dose levels in participants with lupus nephritis	Phase 1	Completed (January 2018)
Study of the combination of ixazomib and fulvestrant	To determine the proper dose and side effects of ixazomib when combined with fulvestrant	Phase 1	Completed (June 2018)
Dose-finding, pharmacokinetics, and safety of meropenem-vaborbactam in pediatric patients with bacterial infections	To determine the dose-finding and evaluate pharmacokinetics, safety, and tolerability in pediatric patients (˂18 years) with serious bacterial infections	Phase 1	Ongoing
Cumulative irritation test of tavaborole	To determine the cumulative irritation potential of tavaborole	Phase 1	Completed (February 2007)
Study to assess local tolerability of crisaborole 2% ointment in healthy participants	To estimate observed application site adverse events following topical applications of crisaborole and vehicle in healthy participants	Phase 1	Completed (April 2019)
Combination therapy with bortezomib with/without venetoclax in multiple myeloma	To study side effects and best dose of venetoclax combined with daratumumab, bortezomib, and dexamethasone in relapsed or refractory multiple myeloma	Phase 1 Phase 2	Ongoing
Combination therapy with bortezomib in plasma cell leukemia	To determine the MTD/RD of bortezomib and pegylated liposomal doxorubicin hydrochloride when in combination with a fixed dose of daratumumab, lenalidomide, and dexamethasone (phase 2)	Phase 1 Phase 2	Completed (December 2018)
Combination chemotherapy with bortezomib in lymphoma	To determine the safety and the MTD of bortezomib when combined with rituximab, methotrexate, and cytarabine in lymphoma	Phase 1 Phase 2	Ongoing
Ixazomib in the prophylaxis of chronic graft-versus-host disease	To evaluate the efficacy of ixazomib at the doses stablished in phase 1	Phase 1 Phase 2	Ongoing
PS-341 ^8^ followed by removal of prostate in prostate cancer	To study the effect of systemic treatment with PS-341 ^8^ doing correlative scientific markers assessing apoptosis; to evaluate protease protein targets and angiogenesis markers	Phase 2	Completed (December 2013)
Bortezomib and vorinostat in patients with multiple myeloma who have undergone stem cell transplant	To evaluate the toxicity of the use of vorinostat and bortezomib as maintenance therapy after autologous transplant	Phase 2	Completed (March 2017)
Fulvestrant with/without bortezomib in inoperable locally or metastatic estrogen receptor positive breast cancer	To determine whether the combination of bortezomib to fulvestrant improves median progression free survival	Phase 2	Ongoing
Bortezomib and lenalidomide in mantle cell lymphoma	To determine the complete and partial response rate of bortezomib and lenalidomide therapy in relapsed or refractory mantle cell lymphoma	Phase 2	Completed (April 2019)
Combination therapy with bortezomib in previously untreated symptomatic multiple myeloma	To evaluate the complete and near-complete response rate of the bortezomib/pegylated liposomal doxorubicin regimen in previously untreated, symptomatic multiple myeloma	Phase 2	Completed (September 2019)
Rituximab, venetoclax, and bortezomib in lymphoma	To determine the overall response rate of rituximab, venetoclax, and bortezomib in relapsed/refractory diffuse large B-cell lymphoma	Phase 2	Ongoing
Combination therapy with/without bortezomib and dexamethasone in immunoglobulin light chain amyloidosis	To evaluate the overall hematologic response rate for ibrutinib with bortezomib and dexamethasone added for lack of response in amyloid light chain	Phase 2	Completed (April 2019)
Combination therapy with bortezomib followed by combination therapy with ixazomib in multiple myeloma	To evaluate the progression-free survival of patients with daratumumab, bortezomib, and dexamethasone treatment followed by daratumumab, ixazomib, and dexamethasone treatment	Phase 2	Ongoing
Melphalan and bortezomib as conditioning regimen for autologous and allogeneic stem cell transplants in multiple myeloma	To evaluate the effectiveness of bortezomib when combined to standard chemotherapy medicine(s) for treatment of multiple myeloma	Phase 2	Completed (May 2019)
Study of ixazomib and erlotinib in solid tumors	To find the MTD of the combination of ixazomib and erlotinib in advanced solid tumors; to study the safety of these drugs	Phase 2	Ongoing
Efficacy and safety of ixazomib and dexamethasone refractory autoimmune cytopenia	To evaluate the safety and efficacy of this combination in refractory autoimmune cytopenia	Phase 2	Completed (October 2019)
Ibrutinib and ixazomib citrate in relapsed or refractory Waldenstrom macroglobulinemia	To study the side effects of ibrutinib citrate when combined with ixazomib; to determine the effect in refractory Waldenstrom macroglobulinemia	Phase 2	Ongoing
Ixazomib for Desensitization	To study the effect of ixazomib to desensitize highly sensitized kidney transplant recipients	Phase 2	Ongoing
Absorption and systemic study of tavaborole in patients with moderate to severe onychomycosis (ADME ^9^ I)	To determine the absorption, systemic pharmacokinetics and accumulation in the nail of tavaborole	Phase 2	Completed (May 2007)
Absorption and systemic study of tavaborole in patients with moderate to severe onychomycosis (ADME II)	To determine the absorption and systemic pharmacokinetics of tavaborole	Phase 2	Completed (May 2007)
Safety and efficacy study of crisaborole ointment to treat plaque type psoriasis	To determine the safety and efficacy of crisaborole ointment 5% in the treatment of plaque type psoriasis	Phase 2	Completed (March 2008)
Crisaborole ointment 2% skin biomarker biopsy study in atopic dermatitis	To characterize the mechanism of action of crisaborole ointment 2% through evaluation of efficacy and changes in key skin biomarkers in atopic dermatitis lesions treated with crisaborole ointment 2%	Phase 2	Completed (May 2018)
Combination therapy with/without bortezomib in previously untreated multiple myeloma	To study the effect of lenalidomide, dexamethasone, and bortezomib compared to dexamethasone and lenalidomide alone in treating patients with previously untreated multiple myeloma	Phase 3	Ongoing
Combination therapy with bortezomib in newly diagnosed acute myeloid leukemia	To study the effect of bortezomib and sorafenib tosylate in newly diagnosed acute myeloid leukemia	Phase 3	Ongoing
Ixazomib citrate, lenalidomide, dexamethasone, and zoledronic acid or zoledronic acid alone after radiation therapy in solitary plasmacytoma of bone	To evaluate the impact of combination of ixazomib, lenalidomide, and dexamethasone to zoledronic acid in multiple myeloma progression rate	Phase 3	Ongoing
Study of dexamethasone plus ixazomib or physician’s choice in relapsed or refractory systemic light chain amyloidosis	To determine if this combination therapy improves hematologic response, heart and kidney deterioration and mortality rate comparing to a physician’s choice of a chemotherapy regimen in relapsed or refractory systemic light chain amyloidosis	Phase 3	Ongoing
Study of oral ixazomib maintenance therapy after initial therapy in newly diagnosed multiple myeloma not treated with stem cell transplantation	To determine the effect of ixazomib maintenance therapy on progression-free survival in newly diagnosed multiple myeloma who have had a major or partial response to initial therapy and who have not undergone stem-cell transplantation	Phase 3	Ongoing
Efficacy, safety, tolerability of meropenem-vaborbactam compared to best available therapy in treating serious infections in adults	To compare the efficacy, safety and tolerability of meropenem-vaborbactam with the best available therapy for infections caused by carbapenem-resistant *Enterobacteriaceae*	Phase 3	Completed (July 2017)
Efficacy and safety evaluation of tavaborole topical solution to treat onychomycosis of the toenail	To determine whether tavaborole topical solution is a safe and effective treatment for onychomycosis of the toenail	Phase 3	Completed (January 2013)
Evaluation of the safety and pharmacokinetics of tavaborole topical solution for the treatment of onychomycosis in children and adolescents	To evaluate the safety and pharmacokinetics of tavaborole 5% topical solution in treating distal subungual onychomycosis of the toenail in children and adolescents	Phase 4	Completed (July 2017)

^1^ CHOP: Cyclophosphamide, doxorubicin, vincristine, prednisolone; ^2^ BCL-XS: Small B-cell lymphoma; ^3^ MTD: Maximum tolerated dose; ^4^ AMG-655: Conatumumab; ^5^ AIDS: Acquired immune deficiency syndrome; ^6^ RD: Recommended dose; ^7^ ART: Antiretroviral therapy; ^8^ PS-341: bortezomib; ^9^ ADME: Absorption, Distribution, Metabolism and Elimination (pharmacokinetic parameters).

**Table 2:** Growth inhibition (IC_50_^1^, µM) effect of chalcone-boronic acids (**2**, **4**, and **5**) and chalcone-carboxylic acids (**9** and **10**) analogues in breast cancer cell lines.

Compounds	MDA-MB-435	MDA-MB-231
**2**	10	8.8
**4**	16	8.5
**5**	8.8	8.8
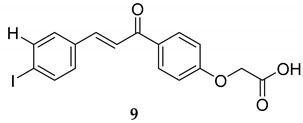	18	44
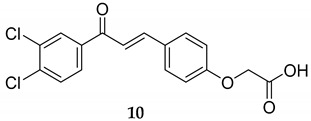	13	18

^1^ IC_50_: Half maximal inhibitory concentration.

**Table 3:** Antiproliferative activity of chalcone-boronic acid analogue **36** in different cell lines.

Cell Line	GI_50_^1^ (µM)
MCF7 (breast cancer)	0.034
SNB-19 (CNS ^2^ cancer)	≤0.01
NCI-H460 (lung cancer)	0.028
SNB-19 (colon cancer)	≤0.01
UACC-62 (melanoma)	0.034
SK-OV-3 (ovarian cancer)	≤0.01
DU-145 (prostate cancer)	0.050

^1^ GI_50_: Half maximal cell growth inhibitory concentration; ^2^ CNS: Central nervous system.

**Table 4:** Effects on growth inhibition (IC_50_, µM**)** of fulvestrant and compound **43** in different breast cancer cell lines.

**Compound**	**MCF-7**	**T47D**
Fulvestrant 43	0.0015 0.0032	0.0012 0.0061

**Table 5:** Minimum inhibitory concentration (MIC, µg/mL) of β-lactam antibiotics alone and when combined with compound **75**.

**CPM ^1^**	**CPM + 75**	**CTLZN ^2^**	**CTLZN + 75**	**MP ^3^**	**MP + 35**
32	≤0.06	32	0.125	1	≤0.015

^1^ CPM: cefepime; ^2^ CTLZN: ceftolozane; ^3^ MP: meropenem.

**Table 6:** Minimum modulatory concentration (MMC, µg/mL) of ciprofloxacin and norfloxacin when alone or combined with compounds **76** or **77**.

**CIPR ^1^**	**CIPR + 76**	**CIPR + 77**	**NOR ^2^**	**NOR + 76**	**NOR + 77**
16	4	4	64	8	8

^1^ CIPR: ciprofloxacin; ^2^ NOR: norfloxacin.

**Table 7:** Inhibitory activity (K*i*, µM) of compound **105** in different flaviviral proteases.

**Dengue Virus Protease**	**West Nile Virus Protease**	**Zika Virus Protease**
0.051	0.082	0.04

**Table 8:** Examples of known biological activities of benzoxaborole compounds.

Compound	Biological Activity	References
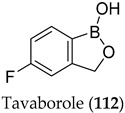	Drug for treatment of onychomycosis—leucyl-tRNA synthetase inhibitor	[88,90]
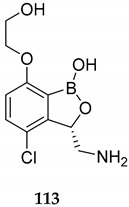	Selective leucyl-tRNA synthetase inhibitor for *Mycobacterium tuberculosis* (*M. tuberculosis*, IC_50_^1^ of 0.2 μM)	[91]
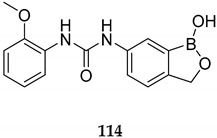	Selective inhibitor of human carbonic anhydrases (CAs) (K*i* ^2^ of 69 -414 nM)	[92]
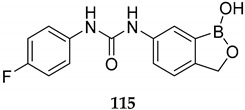	Selective inhibitor of β-CA (*Cryptococcus neoformans*, K*i* of 78 nM; *Candida glabrata*, K*i* of 75 nM)	[93]
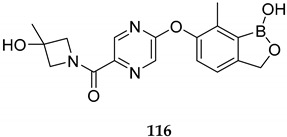	Anti-*Plasmodium* agent (*P.* *falciparum*, ED_90_^3^ of 0.85 mg/kg) - promising for the treatment of malaria	[94]
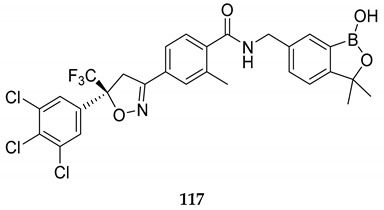	Oral ectoparasiticide agent for *Dermacentor variabilis* (American dog ticks) and *Ctenocephalides felis* (cat fleas) (EC_50_^4^ of 96.6 μM)	[95]
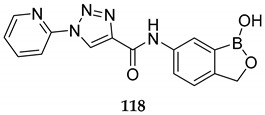	Topical agent for treatment of cutaneous leishmaniasis (*Leishmania major*, EC_50_ of 4.26 μM; *L.tropica*, EC_50_ of 2.01 μM)	[96]
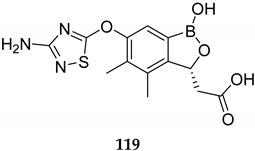	Serine β- lactamase inhibitor (Class A, IC_50_ ˂ 0.005–0.026 μΜ; Class C, IC_50_ of 0.012–0.067 μM; Class D, IC_50_ of 0.088–0.12 μM) of pathogens such as *E. coli* (MIC ^5^ of 0.25–1 μg/ml)	[97]
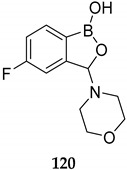	Antiproliferative and proapoptotic agent for ovarian cancer line cell A2780 (IC_50_ of 14.8 μM)	[98]
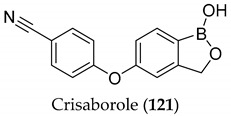	Topical anti-inflammatory drug for atopic dermatitis—phosphodiesterase type 4 (PDE4) inhibitor promising for psoriasis	[99]

^1^ IC_50_: Half maximal inhibitory concentration; ^2^ K*i*: Inhibitory constant; ^3^ ED_90_: Minimum effective dose in 90% of patients; ^4^ EC_50_: Half maximal effective concentration; ^5^ MIC: Minimum inhibitory concentration.
